# Monomicelle‐Directed Engineering of Strained Carbon Nanoribbons as Oxygen Reduction Catalyst

**DOI:** 10.1002/advs.202302930

**Published:** 2023-06-29

**Authors:** Dongping Xue, Yingying Guo, Bang‐An Lu, Huicong Xia, Wenfu Yan, Dongfeng Xue, Shichun Mu, Jia‐Nan Zhang

**Affiliations:** ^1^ School of Materials Science and Engineering Zhengzhou University Zhengzhou 450001 P. R. China; ^2^ State Key Laboratory of Inorganic Synthesis and Preparative Chemistry Jilin University Changchun 130012 P. R. China; ^3^ Multiscale Crystal Materials Research Center Institute of Advanced Materials Science and Engineering Shenzhen Institute of Advanced Technology Chinese Academy of Science Shenzhen 518055 P. R. China; ^4^ State Key Laboratory of Advanced Technology for Materials Synthesis and Processing Wuhan University of Technology Wuhan 430070 P. R. China

**Keywords:** carbon nanoribbons, electrocatalysis, monomicelle‐directed engineering, oxygen reduction reaction, strain effect

## Abstract

To date, precisely tailoring local active sites of well‐defined earth‐abundant metal‐free carbon‐based electrocatalysts for attractive electrocatalytic oxygen reduction reaction (ORR), remains challenging. Herein, the authors successfully introduce a strain effect on active C–C bonds adjacent to edged graphitic nitrogen (N), which raises appropriate spin‐polarization and charge density of carbon active sites and kinetically favor the facilitation of O_2_ adsorption and the activation of O‐containing intermediates. Thus, the constructed metal‐free carbon nanoribbons (CNRs‐C) with high‐curved edges exhibit outstanding ORR activity with half‐wave potentials of 0.78 and 0.9 V in 0.5 m H_2_SO_4_ and 0.1 m KOH, respectively, overwhelming the planar one (0.52 and 0.81 V) and the N‐doped carbon sheet (0.41 and 0.71 V). Especially in acidic media, the kinetic current density (*J_k_*) is 18 times higher than that of the planar one and the N‐doped carbon sheet. Notably, these findings show the spin polarization of the asymmetric structure by introducing a strain effect on the C–C bonds for boosting ORR.

## Graphical abstract

The curved edge‐rich N‐doped CNRs (CNRs‐C) are successfully achieved by a monomicelle‐directed assembly approach. The unique atomic‐scaled strain modulation can induce spin‐polarization optimization on C active sites adjacent to the edge graphitic N, thus accelerating surface ORR kinetics.

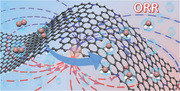

## 1 Introduction

Earth‐abundant metal‐free carbon‐based catalysts (MCCs) have been extensively investigated as potential alternatives to precious commercial Pt/C catalysts for oxygen reduction reaction (ORR) in fuel cells and Zn‐air batteries, owing to their low‐cost, anti‐Fenton reactivity, and excellent stability.^[^ ^1^, ^2^, ^3^, ^4^, ^5^ ^]^ However, attempts to synthesize high efficient MCCs so far had limited success. Charge and spin are two intrinsic properties of electrons, so a highly promising strategy is the charge transfer/redistribution on the *π*‐conjugated carbon atoms in terms of creating defects. There are two types of defects, including heteroatom doping^[^ ^6^, ^7^, ^8^, ^9^, ^10^, ^11^ ^]^ and structural defects into the carbon skeleton^[^ ^6^, ^12^, ^13^ ^]^ which endow the surrounding carbon atoms with catalytic activity.^[^ ^14^, ^15^ ^]^

Considering that constructing defect‐rich sites in carbon is the key point to further improve the activity of MCCs, as demonstrated in the different catalysts of 1D graphene nanoribbons^[^ ^16^ ^]^ and vertical carbon nanotube arrays.^[^ ^17^ ^]^ Among them, carbon nanoribbons (CNRs) are one of the most attractive materials owing to salient large aspect ratios and numerous defects along edges.^[^ ^18^, ^19^ ^]^ A great number of efforts have been devoted to the preparation of CNRs by plasma technique,^[^ ^19^, ^20^, ^21^ ^]^ chemical etching,^[^ ^16^ ^]^ and chemical vapor deposition.^[^ ^22^ ^]^ However, CNRs exhibit atomically thin and smooth edge defects with the high symmetry of graphite carbon hexagons and *π* electronic conjugation structures, which can prevent a sizable electronic bandgap from formation and degradation of electrochemistry properties.^[^ ^23^ ^]^ The change in the electronic spin multiplicity state between reactants and products would be the origin of the high reaction barriers for and slow reaction kinetics of the ORR.^[^ ^24^, ^25^ ^]^ Therefore, the restrictions of the spin conservation rule must be overcome to lower the reaction barrier and accelerate the ORR, to obtain MCCs comparable to metal‐containing electrocatalysts in acidic media, that is, promote the spin polarization of the active site to improve the adsorption/desorption behavior of reaction intermediates on the catalyst surface.

Recent research has revealed that the strain effects on 2D carbon materials can significantly boost their electrocatalytic performance by electron redistribution.^[^ ^26^ ^]^ Such as the curved single atomic FeN_4_‐C carbon materials were obtained from a spiral structural metal‐organic framework (MOF) parent precursor.^[^ ^27^, ^28^, ^29^, ^30^ ^]^ In this regard, CNRs with rich edge defects and controllable curvature are highly expected. So far, the curvation controlling of carbon surfaces is only achieved by increasing pyrolysis temperatures, where the decarbonization and C–C bond rearrangement simultaneously occur at high temperatures. Unfortunately, the CNRs structure is probably uncontrollable at high pyrolytic temperatures. Therefore, to date, very little success has been achieved in precisely modulating CNRs' curved edge‐defect sites due to the lack of molecule level parent.

The monomicelle‐directed method, which involves the triblock polymer assembly process, is an ideal strategy to contribute to the 1D nanoarchitectures, such as nanowires with tunable manipulation.^[^ ^31^ ^]^ The supramolecular block triblock polymer/surfactant and precursor species can form the smallest micelle monomer unit‐ “monomicelle” through hydrogen bonds, coulombic and/or other noncovalent interactions. Under the strong repulsive force between the micelles, the composite monomers “end‐on” are orderly arranged and partially fused.^[^ ^32^, ^33^ ^]^ Thus, herein we for the first time perform a proof‐of‐concept study by constructing the curved edge‐rich defective carbon nanoribbons (CNRs‐C) via a monomicelle‐directed assembly method using melamine (M) as N and C sources, triphenylphosphine (TPP) as phosphorus (P) sources, and triblock polymer pluronic P123 as surfactant, respectively. The as‐prepared CNRs‐C possess the high surface curvature of the kelp‐like convex structure arranged along the nanoribbons horizontally with ≈5% strain, calculated by C‐C atom pair distances detected with the pair distribution function (PDF) measurement and analysis. The unique strained atomic‐scaled strain effect on the modulation of spin‐polarization for active C sites adjacent to the edge graphitic N is achieved, thus accelerating surface ORR kinetics. Therefore, CNRs‐C, serving as an ORR electrocatalyst, achieves higher activity than carbon nanosheets (CNSs) and nanoribbons with relatively smooth surfaces (CNRs‐S) both in acidic and alkaline media. As a practical application, CNRs‐C presents a super power density for proton exchange membrane fuel cells and for Zn‐air batteries.

## 2 Results and Discussion

### 2.1 Synthesis and Structural Characterization

**Figure 1:**
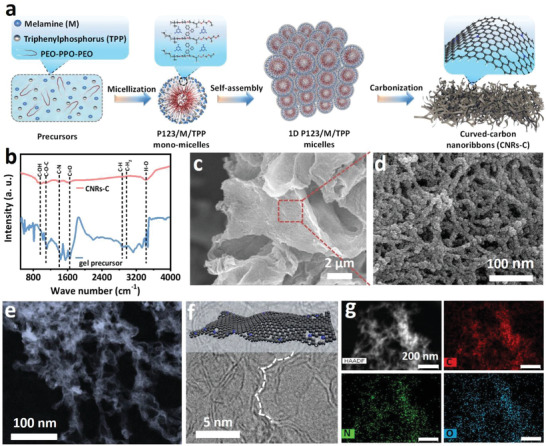
a) Schematic of CNRs‐C formation. b) FTIR spectra of the gel precursor (before pyrolysis) and CNRs‐C. c,d) SEM, e) HAADF‐STEM, and f) HRTEM images of CNRs‐C. g) HAADF‐STEM image of CNRs‐C and the corresponding elemental mapping images of C, N, and O.

CNRs‐C were synthesized by monomicelle‐directed confinement (**Figure** **1**a). Typically, the 1D M/TPP prepolymer forms by self‐assembly of composite monomicelles under the confinement of the micelle matrix (pluronic P123/TPP/M monomicelles) after the evaporation of the preferential solvent (alcohol/water) at 80 °C overnight, resulting in a dry gel containing pluronic P123, M, and TPP. As expected, in the gel, the hydrophilic M molecules were anchored over the polyethylene oxide (PEO) shell of the pluronic P123 micelles via hydrogen bonds and the hydrophobic TPP molecules were confined in the poly(propylene oxide) (PPO) core (Figure 1b, in the FTIR spectrum, the characteristic peak at 2863 cm^−1^ in the gel precursor is saturated C‐H). Subsequently, 1D edge‐defect‐rich CNRs‐C were formed by removing the surfactant and P‐doping sites after carbonization in argon (Ar) at 1000 °C for 6 h at a heating rate of 10 °C min^−1^.

Scanning electron microscopy (SEM) images (Figure 1c,d) clearly showed that the 2D interwoven nanoarchitecture consists of well‐defined nanoribbon networks with stacked porous structures. Notably, the high‐angle annular dark‐field scanning transmission electron microscopy (HAADF‐STEM) image clearly revealed the edge‐curved CNRs‐C (Figure 1e,f, and Figure S1, Supporting Information). In addition, Figure 1f revealed that sp^2^‐hybridized carbon may be dominant, indicating that CNRs‐C has a highly graphitic structure.^[^ ^34^ ^]^ Defective carbon nanoribbons with excellent charge (electron and ion) transport capabilities are thought to promote the efficient exposure of more accessible active sites. The energy‐dispersive X‐ray spectroscopy (EDS) mapping images of CNRs‐C (Figure 1g and Figure S2, Supporting Information) showed the presence and uniform distribution of the C, N, and O in the nanoarchitecture. Notably, no P signal was detected, indicating that dephosphorization occurs at a high pyrolysis temperature (1000 °C). Moreover, X‐ray photoelectron spectroscopy (XPS) for P 2p of the 1D micelles obtained at different pyrolysis temperatures (600, 800, and 1000 °C) confirmed this result (Figure S3, Supporting Information). With increasing pyrolysis temperature, the P 2p signal became weaker until it completely disappeared at 1000 °C. The negligible single P peak reveals that P removal at high pyrolysis temperatures likely generates topological defects, such as pentagonal and heptagonal defects, during the partial reconstruction of dangling bonds,^[^ ^35^ ^]^ further enhancing the catalytic performance.

**Figure 2:**
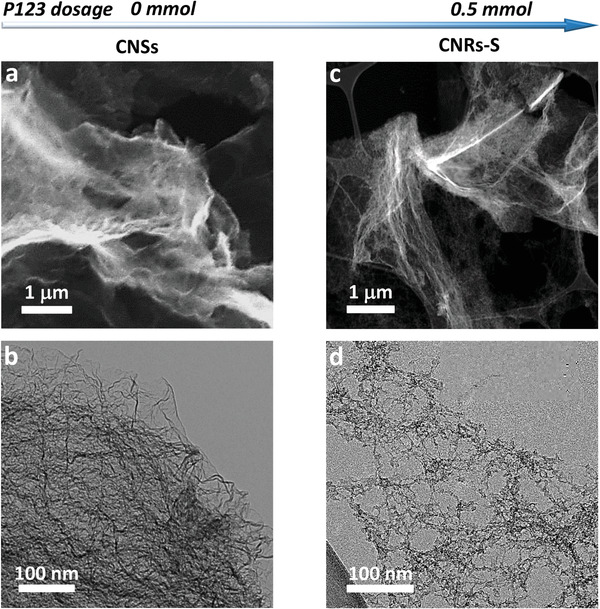
SEM images of a) CNSs (prepared without surfactant) and c) CNRs‐S (prepared with 0.5 mmol of surfactant). TEM images of b) CNSs and d) CNRs‐S.

To confirm the role of the 1D nanoribbon structure and strain effect in enhancing the ORR performance, controlled experiments were performed to synthesize different morphologies of carbon‐based catalysts using a protocol similar to that for CNRs‐C, but varying the pluronic P123 content. Additionally, carbon nanosheets (CNSs) were prepared in the absence of pluronic P123. The SEM (Figure S4, Supporting Information), STEM (**Figure** **2**a), and TEM (Figure 2b) images revealed that CNSs are composed of 2D successive sheets with a wrinkled surface of approximate dimensions of 10 × 10 µm and thickness of 2–4 nm. CNRs with relatively smooth edges (CNRs‐S) were prepared by adding 0.5 mmol of pluronic P123 (Figure 2c,d). Thus, pluronic P123 plays a key role in the formation of edge‐defect‐rich 1D nanoribbons and in developing a curvature‐surfactant templating‐dependent relationship.

**Figure 3:**
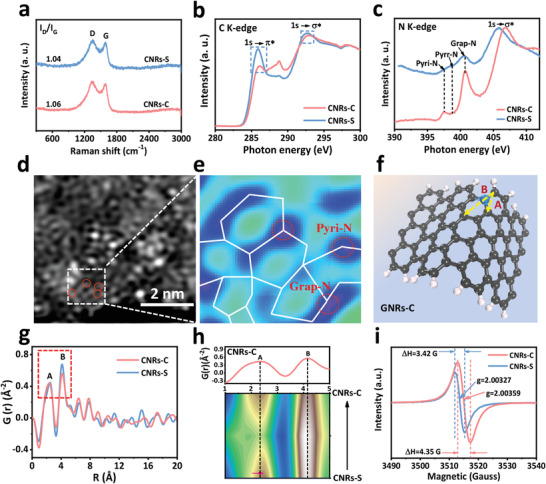
a) Raman spectra of CNRs‐S and CNRs‐C. b) C K‐edge and c) N K‐edge XANES spectra of CNRs‐S and CNRs‐C. d) HAADF‐STEM image of CNRs‐C. e) Expanded image of the dotted box in d, presenting the pyri‐N and grap‐N sites at the edge. f) The typical A and B labels of peaks correspond to the local atomic pair distances in the atomic structure of CNRs‐C. g) PDF pattern for CNRs‐C and CNRs‐S. h) Expanded PDF pattern of the dotted box in (g). i) ESR spectra of CNRs‐C and CNRs‐S.

The X‐ray diffraction (XRD) patterns of CNRs‐C, CNRs‐S, and CNSs exhibited the characteristic peaks of the (002) and (101) planes (Figure S5, Supporting Information), demonstrating their highly graphitic structures. As expected, the Raman spectra indicated that CNRs‐C, CNRs‐S, and CNSs exhibited maximum *I_D_*/*I_G_* values of 1.06, 1.04, and 1.03 (**Figure** **3**a and Figure S6, Supporting Information), respectively, indicating that the unique CNRs‐C has a higher degree of intrinsic defects (such as edges and pentagonal defects) in the carbon matrix.^[^ ^36^ ^]^ The XPS spectrum for C 1s revealed the dominance of sp^2^‐hybridized carbon, further confirming that CNRs‐C has a highly graphitic structure (Figures S7 and S8, Supporting Information). The N1s spectrum of CNRs‐C exhibited peaks associated with pyridinic N (pyri‐N, 397.85 eV), pyrrolic N (pyrr‐N, 398.61 eV), graphitic N (grap‐N, 400.59 eV), and intercalated N molecules and/or oxides (N–O, 402.88 eV) (Figure S9 and Tables S1 and S2, Supporting Information), indicating the successful doping of 3.56 at% N. Among these peaks, the grap‐N domains are the N‐dopant type, which are very beneficial for improving the acid tolerance of the CNRs‐C catalyst in addition to its high graphitization.^[^ ^37^ ^]^ The Brunauer–Emmett–Teller (BET) and pore size distribution analyses (Figure S10 and Table S3, Supporting Information) show that CNRs‐C, CNRs‐S, and CNSs have similar and large specific surface areas and pore size distribution (mainly mesoporous), which is conducive to exposing active site and mass transfer and improving catalytic activity.

To accurately analyze the regular changes in the carbon structure of the synthesized samples, the normalized C K‐edge X‐ray absorption near‐edge structure (XANES) spectra are shown in Figure 3b. The sharp peak observed at ≈285.5 eV is attributed to the C 1s→π* transition, which is indicative of the integrity level of the sp^2^‐hybridized carbon configuration.^[^ ^34^, ^38^ ^]^ For CNRs‐C, the intensity of the π* state was significantly lower than that for CNRs‐S, suggesting that partial edge‐defect reconstruction occurred for CNRs‐C, inducing more non‐hexagonal defects, which slightly damage the entire π*conjugation.^[^ ^39^ ^]^ The σ* state observed at ≈292.7 eV is sensitive to distortions and strain of the carbon bonds in the hexagonal graphitic structure. Notably, the characteristic peak associated with σ* states shifts toward higher energies for CNRs‐S relative to CNRs‐C, indicating an increase in the carbon lattice strain in the sp^2^ rings.^[^ ^40^ ^]^

In addition, the N K‐edge XANES spectra detected pyri‐N, pyrr‐N, and grap‐N structures (Figure 3c).^[^ ^41^ ^]^ After thermal treatment, the grap‐N species was dominant in CNRs‐C and CNRs‐S. Furthermore, the hexagonal structure of the N‐doped carbon substrate was clearly observed by HAADF‐STEM (Figure 3d and Figure S11a, Supporting Information). In particular, the corresponding inverse fast Fourier transform (IFFT) images (Figure 3e and Figure S11b, Supporting Information) indicated that grap‐N and pyri‐N are present in the sample. Moreover, the number of grap‐N sites was greater than that of pyri‐N sites at the edge of the sample, which is consistent with the XPS and XANES results. Simultaneously, the IFFT image showed that compared with CNRs‐S (Figure S11, Supporting Information), the carbon‐edged hexagonal structure of CNRs‐C was distorted more significantly, further confirming that the nanoribbons constituting the CNRs‐C catalyst are subjected to a strain effect at the edges.

PDF analysis was conducted to determine the atomic‐level structures of the catalysts. The PDF of a material can be obtained experimentally by the Fourier transformation of the Bragg scattering pattern (i.e., diffraction), providing direct‐spatial insight into any long‐range ordered structure. The presence of short‐range structures based on diffuse scattering intensity (for the detailed analysis procedure, see the Supporting Information)^[^ ^42^, ^43^ ^]^ and the corresponding Rietveld analyses are shown in Figure 3g,h. A similar PDF curve was observed for CNRs‐C and CNRs‐S (Figure 3h). The 2D profile corresponding to the two main peaks (A and B) of CNRs‐C had the darkest color, and the position of these peaks shifted to the left relative to CNRs‐S (Figure 3h), confirming that the atomic C–C pair distance in CNRs‐C was further shortened and the strain was intensified. For the two main peaks A and B, the C–C atom pair distances for CNRs‐C were 2.32 and 4.07 Å, respectively, which is 5.81% and 4.44% shorter than those for the theoretical planar carbon hexagonal structure (2.46 and 4.26 Å, respectively). For convenience in later simulation calculations, the average shortening distance was selected as 5%. Therefore, the peaks of CNRs‐C labeled A and B, may be assigned to the C–C atomic pair distances at a strain of −5% (as indicated by the A and B labels in the model for edge‐defect‐rich curved N‐doped CNRs at 5% strain, inset in Figure 3f). With an increase in pyrolysis temperature and the presence of structure‐directing agents, the C–C active site with strain and an edge‐defect‐rich structure was successfully realized for CNRs‐C, originating from structural reconstruction by volatilization of the small molecules and dephosphorization at 1000 °C to degrade the carbon hexagonal structure.

Electron paramagnetic resonance (ESR) spectroscopy was conducted to reveal the electron spin structure of the nanoribbons (Figure 3i). Notably, the ESR spectra showed a distinct shift and signal reinforcement when the degree of strain was increased. It is well known that an unpaired electron that gains or loses angular momentum alters the g‐factor value.^[^ ^44^ ^]^ Thus, the degree of strain is beneficial for modulating the electron spin configuration and chemical environment of the carbon sites. With an increase in the degree of strain, the width (ΔH) and intensity of the spectral lines increased significantly, revealing that CNRs‐C has a higher spin density, which results in the spin polarization of electrons at the active C sites, thereby increasing the magnetic moment. This large magnetic moment may contribute to the catalytic activity of CNRs‐C because magnetic moments are related to electron motion and transfer.^[^ ^10^ ^]^

### 2.2 Formation Mechanism

Translucent white gels were first generated after evaporation at 80 °C (Figure S12, Supporting Information), indicating that the precursor was assembled with surfactants. The gels were composed of amphiphilic Pluronic P123/M/TPP spherical composite monomicelles with PPO segments as the core and precursor‐associated PEO segments as the shell. Fourier‐transform infrared (FTIR) spectroscopy of the gel precursor showed an H‐O characteristic peak at 3423 cm^−1^ (Figure 1b), which confirms that the surfactants and precursors form monomicelles via hydrogen bonds.

**Figure 4:**
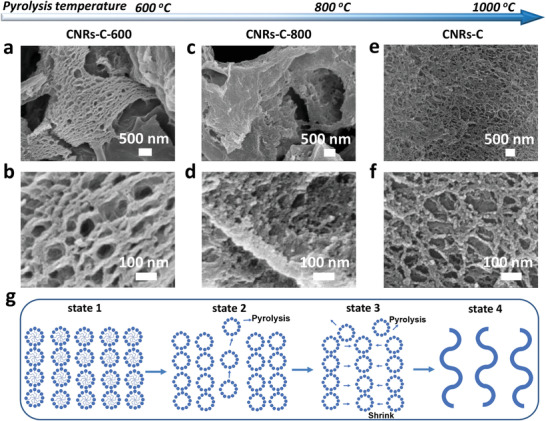
SEM images of a,b) CNRs‐C‐600, c,d) CNRs‐C‐800, and e,f) CNRs‐C at different magnifications. g) Schematic of the structural evolution of CNRs pyrolyzed at different temperatures.

In the subsequent heating pyrolysis process for surfactant removal, the carbon layer formed at 600 °C had a groove‐like structure (**Figure** **4**a and Figure S13a, Supporting Information). The high‐magnification SEM image (Figure 4b) clearly showed the continuous pearl chain‐like channel structure with a wall thickness of ≈50 nm, indicating that the spherical monomicelles are arranged in an orderly manner and are partially crosslinked and that the micelle core is removed to form a hole with a diameter of ≈100 nm.

When the pyrolysis temperature was increased to 800 °C, the carbon layer exhibited abundant folds (Figure S13b, Supporting Information and Figure 4c). The magnified SEM image (Figure 4d) clearly showed that these folds are composed of partially separated 1D nanostructures with diameters of ≈20 nm, indicating that the pearl chain‐like shells disintegrated into densely aligned nanoribbons. Owing to the small amount of organic polymer and the inability of the pearl chain‐like shell template to split completely, the formed nanoribbons were not completely independent and had a large diameter.

When the pyrolysis temperature was increased to 1000 °C, completely separated kelp‐like CNRs with diameters of ≈10 nm were achieved (Figure 4e,f and Figure S13c, Supporting Information). This suggests that the curved structures of the nanoribbons originate from the interior wall structures of the pearl chain‐like channels that remain after partial fusion of the monomicelles. In particular, the nanoribbons are clearly separated from each other, which is likely owing to the evaporation of the heteroatom‐doping sites. Thus, the aggregation and broadening of the growing nanoribbons were prevented. Based on the FTIR spectra, the saturated C‐H characteristic peak at 2863 cm^−1^ in the gel precursor disappeared in CNRs‐C (Figure 1b). The remaining peaks in the gel precursor were observed for CNRs‐C, but the peak intensity was significantly weakened, which confirms that the carbon structure is partially reconstructed by the removal of heteroatoms during pyrolysis. In addition, only slightly folded nanosheets were formed using the initial precursor solution without monomicelles (Figure S5, Supporting Information), indicating that monomicelle assembly is required for the formation of the curved interwoven nanoribbons.

Based on these observations, a monomicelle‐directed confinement process is proposed for the formation of curved CNRs (Figure 4g). Initially, Pluronic P123/M/TPP spherical composite monomicelles are formed via alcohol/water evaporation.^[^ ^32^ ^]^ Subsequently, the composite monomicelles begin to assemble and further crosslink with the M/TPP prepolymer. The formed composite monomicelles repel each other owing to their similar electrical properties, which drives the “end‐on” arrangement of composite monomicelles, resulting in the partial fusion of the monomicelles.^[^ ^45^ ^]^ Therefore, pearl chain‐like channels are formed instead of cylindrical channels (state 1). With an increase in the pyrolysis temperature, the core of the monomicelle template is removed, forming a pearl chain‐like shell (state 2). In addition, as the pyrolysis temperature increases, the carbon layer continuously contracts owing to the volatilization and condensation of organic molecules, leading to matter diffusion of the channel walls (state 3). As matter diffusion proceeds, the channel‐to‐channel distance is further reduced until the isolated channels merge with their nearest neighbors (i.e., wall splitting). Finally, the chain–channel wall evolves into nanoribbons (state 4). Simultaneously, the interior wall structures of the pearl chain‐like channels transform into axially arranged wavy structures, forming the curved morphology of the CNRs. Notably, the flexible space‐filling and steric repulsion of the amphiphilic Pluronic P123 chains between the growing nanoribbons^[^ ^46^ ^]^ effectively prevent the nanoribbons from converging into wider nanoribbons or nanosheets.

### 2.3 Electrochemical ORR Performances

**Figure 5:**
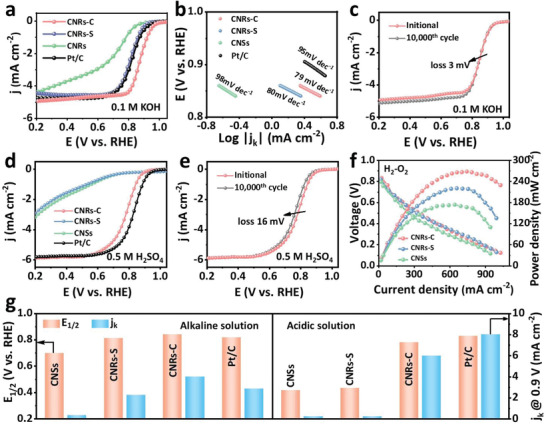
a) LSV curves, b) Tafel slopes, and c) ORR polarization curves (1600 rpm) of CNRs‐C before and after 10 000 cycles in O_2_‐saturated 0.1 m KOH. d) LSV curves in O_2_‐saturated 0.5 m H_2_SO_4_ at 1600 rpm for CNRs‐C, CNRs‐S, CNSs, and Pt/C. e) The ORR polarization curves (1600 rpm) of CNRs‐C before and after 10 000 cycles in O_2_‐saturated 0.5 m H_2_SO_4_. f) Polarization and power density curves of CNRs‐C, CNRs‐S, and CNRs under 2 bar H_2_‐O_2_. g) Comparison of the kinetic current density (*J*_k_) and E_1/2_ of CNRs‐C, CNRs‐S, CNSs, and Pt/C.

The electrocatalytic performance of the CNRs‐C for the ORR was investigated using a rotating disk electrode (RDE) with a mass loading of ≈0.10 mg cm^−1^ in 0.1 m KOH. Linear sweep voltammetry (LSV) curves indicated that CNRs‐C has outstanding ORR activity with an onset potential (*E_onset_*) of 0.95 V versus a reversible hydrogen electrode (RHE) and half‐wave potential (*E*_1/2_) of 0.90 V (vs RHE), which are more positive than those of commercial Pt/C (0.96 and 0.83 V), CNRs‐S (0.93 and 0.81 V), and CNSs (0.89 and 0.71 V) (**Figure** **5**a and Figure S14, Supporting Information). Therefore, strain is crucial for achieving CNRs‐C with high activity. The Tafel slope of CNRs‐C (79 mV dec^−1^) was lower than those of the CNSs (98 mV dec^−1^), CNRs‐S (80 mV dec^−1^), and commercial Pt/C (95 mV dec^−1^) catalysts, indicating faster ORR kinetics (Figure 5b).^[^ ^47^ ^]^ The Koutechy–Levich (K–L) plots obtained from the corresponding LSV curves yielded an electron transfer number (*n*) of ≈4.0 for the CNRs‐C electrode (Figure S15, Supporting Information), indicating excellent ORR electrocatalytic kinetics. The rotating ring‐disk electrode (RRDE) measurements further demonstrated the reduction of oxygen molecules to water via an approximate 4e^−^ pathway (n ≈ 4) for the ORR with low peroxide yields (< 8%) (Figure S15a, Supporting Information). This improves the availability of catalysts for metal‐air batteries and fuel cells. As shown in Figure 5g, the kinetic current density (*J*_k_) (4.1 mA cm^−2^) of CNRs‐C at 0.85 V (vs RHE) was significantly enhanced compared with those of CNSs (0.36 mA cm^−2^), CNRs‐S (2.25 mA cm^−2^), and Pt/C (2.87 mA cm^−2^). The methanol tolerance test showed that a relative current density of 81.50% remained for CNRs‐C, whereas a considerable decrease in current density occurred for Pt/C (Figure S16, Supporting Information), indicating high selectivity of CNRs‐C toward methanol oxidation. Moreover, CNRs‐C exhibited a benchmark chronoamperometry response extending over 40 000 s with an initial current retention of 94.90%, which is significantly better than that of Pt/C (74.01%) (Figure S17a, Supporting Information). The LSV curve of CNRs‐C exhibited negligible activity loss after 10 000 cycles (Figure 5c), whereas it dramatically decreased for the Pt/C catalyst after the stability test (Figure S17b, Supporting Information). The outstanding ORR activity and stability of CNRs‐C (Table S4, Supporting Information) are comparable to those of state‐of‐the‐art MCCs.

The ORR performance of CNRs‐C in 0.5 m H_2_SO_4_ electrolyte was also investigated. The LSV curves of CNRs‐C exhibited the ORR activity with an *E*_1/2_ of 0.78 V (vs RHE) and *J*_k_ of 5.3 mA cm^−2^ (Figure 5d,g). These values are comparable to those of the commercial Pt/C catalyst (*E*_1/2_ = 0.83 V vs RHE, *J*_k_ = 8.1 mA cm^−2^), and higher than those of CNSs and CNRs‐S. The value of n during the ORR catalyzed by CNRs‐C was calculated to be ≈3.93 from the LSV curves obtained at different rotating speeds in the range of 400–2500 rpm under O_2_‐saturated acidic conditions and from the K–L plots (Figure S18, Supporting Information). The RRDE measurements (Figure S19, Supporting Information) showed an approximate 4e^−^ pathway for the ORR, with a small ratio of peroxide species (< 10%). Methanol tolerance can be confirmed by introducing methanol during the ORR. As shown in Figure S20a,b, Supporting Information, when introducing 3 m methanol into 0.5 m H_2_SO_4_ solutions, CNRs‐C retained 80% of the original current density. Additionally, the stability of CNRs‐C for ORR was evaluated by performing chronoamperometric measurements at 0.75 V (vs RHE) (Figure S20c, Supporting Information). After 10 000 cyclic voltammetry scans, no distinct shift in the LSV curve was observed (Figure 5e).

### 2.4 Applications

First, CNRs‐C was assembled into membrane electrode assemblies (MEAs) as ORR catalysts and evaluated in an actual proton exchange membrane fuel cell (PEMFC). It exhibits a peak power density of 272.7 mW cm^−2^ at a catalyst loading of 4 mg cm^−2^ (Figure 5f and Figure S23, Supporting Information), overwhelming the CNRs‐S (222.6 mW cm^−2^) and CNSs (175.7 mW cm^−2^). Although still lower than commercial Pt/C (1375 mW cm^−2^), it was significantly superior to most of the reported metal‐free carbon‐based catalysts (Table S5, Supporting Information). This demonstrates that CNRs‐C is a promising electrocatalyst for low‐cost and durable proton exchange membrane fuel cells.

**Figure 6:**
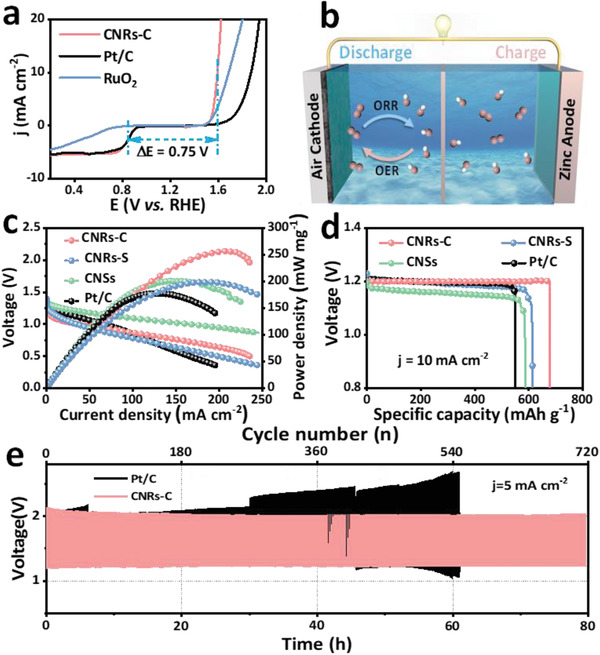
a) LSV curves of CNRs‐C, Pt/C, and RuO_2_ for both the ORR and OER in 0.1 m KOH at 1600 rpm (scan rate: 5 mV s^−1^). b) Schematic of the fabricated zinc‐air battery. c) Polarization and power density curves in a 6 m KOH+0.2 m Zn(CH_3_COO)_2_ electrolyte. d) Discharge curves using CNRs‐C, CNRs‐S, CNSs, and Pt/C as a cathodic catalyst at a current density of 10 mA cm^−2^. e) Discharge‐charge cycling tests of CNRs‐C and Pt/C.

The overall oxygen electrode activity was assessed based on the difference between the oxygen evolution reaction (OER) and ORR metrics. Compared with Pt/C and RuO_2_, CNRs‐C exhibited the smallest *ΔE* (*E_j_*_=10_ − *E*_1/2_) value of 0.75 V (**Figure** **6**a). Based on the outstanding ORR and OER performance, we assembled liquid and flexible all‐solid‐state rechargeable Zn‐air batteries to further evaluate the practical applications of CNRs‐C (Figure 6b).^[^ ^16^, ^48^ ^]^ The CNRs‐C catalyst‐coated carbon paper (≈0.5 mg cm^−2^) and a Zn foil were paired in 6 m KOH as the air cathode and anode, respectively. The polarization curves and corresponding power densities (Figure 6c) showed that CNRs‐C provides a higher output voltage and power density than Pt/C at the same discharge current density in a 6 m KOH+0.2 m Zn(CH_3_COO)_2_ electrolyte. CNRs‐C achieved a maximum mass power density of 259.1 mW mg^−1^ at a current density of 213 mA cm^−2^, which was higher than that of Pt/C (177.5 mW mg^−1^ at a current density of 125 mA cm^−2^). The galvanostatic discharge curves (Figure 6d) indicated that CNRs‐C delivers a higher output voltage (1.2 V) and specific capacity (676.9 mAh g^−1^) than Pt/C (550.7 mAh g^−1^).

As shown in Figure 6e, unlike the Pt/C‐based battery with a distinct voltage change, the CNRs‐C‐based Zn‐air battery provided a lower charge–discharge voltage gap and exhibited excellent stability after 80 h (720 cycles) of continuous operation at a current density of 5 mA cm^−2^. Photographs of the liquid batteries show an open‐circuit voltage (OCV) of 1.394 V and a light‐emitting diode (LED) powered by two liquid Zn‐air batteries with CNRs‐C as the air cathode connected in series (Figure S24, Supporting Information). Therefore, a homemade and scalable all‐solid‐state battery was fabricated, as shown in Figure S25, Supporting Information, which exhibited a surprisingly high OCV of ≈1.445 V and illuminated LED lamps (left to right are blue, red, and yellow) using three all‐solid‐state Zn‐air batteries. The all‐solid‐state batteries exhibited stable charge (1.8 V) and discharge (1.0 V) potentials at a current density of 2 mA cm^−2^ for 6 h, even when the device was bent to 0°, 90°, and 180° (Figure S26, Supporting Information). These results clearly demonstrate the excellent activity and stability of CNRs‐C as a cathodic catalyst for Zn‐air batteries.

### 2.5 Density Functional Theory Calculations

**Figure 7:**
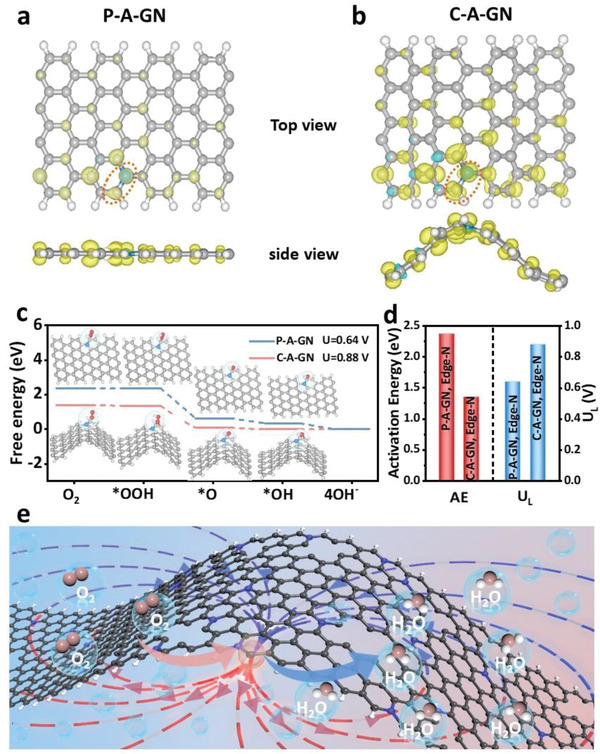
a) P‐A‐GN and b) C‐A‐GN. The isosurface value is 0.0005 eÅ^−3^. c) The free energy of the ORR process of P‐A‐GN and C‐A‐GN. d) The limiting potentials (*U_L_*) of the ORR and activation energy (*AE*) for *OOH dissociation on active C sites in P‐A‐GN and C‐A‐GN. e) Schematic of the ORR of C‐A‐GN.

The Pauli exclusion principle restricts the transfer of an electron from one orbital to its neighbor if an electron of the same spin already occupies that orbital. Thus, the tunneling electrons must be antiparallel to the half‐filled O_2_ π* orbitals. It is known that spin angular momentum is conserved during electron transfer in the catalyst and reactants.^[^ ^49^, ^50^ ^]^ Therefore, density functional theory (DFT) calculations were performed to gain insight into the spin‐activity relationship between the strain and ORR performance. Considering the three types of N‐doping sites (grap‐N, pyri‐N, and pyrr‐N) on the edges of the zig‐zag and armchair defects, six possible models were screened for CNRs with planar carbon structures: grap‐N‐doped (P‐A‐GN)‐, pyri‐N‐doped (P‐A‐N_6_)‐, and pyrr‐N‐doped (P‐A‐N_5_)‐rich armchair and P‐Z‐GN‐, P‐Z‐N_6_‐, and P‐Z‐N_5_‐rich zig‐zag edge carbon defects (Figure S27, Supporting Information). Primarily, the ORR limit potentials (*U_L_*) at these active sites were computed using these models to select the most active N‐dopant defect, which will serve as a guide for the subsequent evaluation of curved edge defect sites (Figure S28 and Table S6, Supporting Information). The P‐A‐GN site exhibited the highest limit potential (0.64 V) and the lowest reaction energy barrier. To gain further insight based on the most active P‐A‐GN site (**Figure** **7**a), the curved grap‐N‐doped armchair edge defects in CNRs with 5% strained C–C atom pairs were constructed (C‐A‐GN, Figure 7b). First, a charge density difference calculation was performed for the P‐A‐GN and C‐A‐GN structures. As shown in Figure S29, Supporting Information, the strain effect causes a change in the electronic structure, and charge transfer occurs from the central C atom to the adjacent N atoms. The Bader charge of C in C‐A‐GN (1.22 e) was larger than that in P‐A‐GN (1.20 e), demonstrating a more distinct charge transfer (Table S7, Supporting Information), which is beneficial for improving the adsorption and desorption of the reaction intermediates under the strain effect.

In addition, the spin distributions of P‐A‐GN and C‐A‐GN were considered. The carbon atoms in P‐A‐GN were not magnetic, with a negligible spin moment (*µ*_B_) (0.053 *µ*_B_); however, the spin moment dramatically increased to 0.238 *µ*_B_ for C‐A‐GN. Therefore, it is demonstrated a linear correlation exists between the spin moment and curvature (Figure S30, Supporting Information), that is, the spin moment increases with increasing curvature of CNRs. Furthermore, as shown in Figure 7a,b, the amount of spin‐charge on the carbon atoms of the active sites in C‐A‐GN is significantly higher than that in P‐A‐GN, which is responsible for the accelerated ORR. Therefore, magnetic nanoribbons have a significant thermodynamic advantage. Thus, the adsorption energies of the ORR species (such as O_2_, *OOH, *O, and *OH) were further calculated (Table S8, Supporting Information). Moreover, the free energy evolution for the ORR and the activation energy for OOH dissociation on the C sites are shown in Figure 7c,d and Figures S31 and S32, Supporting Information.^[^ ^51^, ^52^ ^]^ Notably, the ORR at the C site of C‐A‐GN became exothermic when the electrode potential was lower than the limiting potential (0.88 V), which is higher than that at the C site of P‐A‐GN (0.64 V). Moreover, in the first step, compared to P‐A‐GN, the bond length between carbon sites and *OOH (C‐*OOH) in C‐A‐GN is significantly shortened (shorten from 1.564 to 1.164 Å), and shorter than the bond length of *O‐OH (1.287 Å), fully proving that spin polarization on carbon sites promotes the activation of *OOH and improves 4e^−^ selectivity. Undoubtedly, the strain‐induced changes in the electronic structure such as electron rearrangement and spin polarization significantly reduced the limiting potential and reaction energy barrier of CNRs in the ORR and endowed curved CNRs with an excellent ORR performance (Figure 7e). Therefore, this study demonstrates that by optimizing the spin potentials of catalyst materials, catalysts with populated antibonding *d*‐transition‐metal orbitals are not required to obtain outstanding oxygen electrocatalytic activity.

## 3 Conclusion

Edge‐defect‐rich strained N‐doped CNRs were successfully synthesized using a monomicelle‐directed assembly method. Moreover, the synthesis of carbon nanosheets and nanoribbons with relatively smooth edges can be controlled by adjusting the amount of the monomicelle template. These unparalleled architectures effectively induced charge redistribution and spin polarization on *π*‐conjugated C atoms. More importantly, the strain significantly optimized the activation energy and reaction energy barrier of the oxygen species at the active C sites adjacent to the edge graphitic N, which is key for significantly enhancing the ORR performance. Therefore, the application of CNRs‐C as ORR electrocatalyst yielded excellent ORR performance in both acidic and alkaline media, superior to that of the vast majority of carbon‐based metal‐free catalysts reported to date. In addition, CNRs‐C exhibited an excellent performance when applied in PEMFCs and Zn‐air batteries. Notably, for the Zn‐air battery application, the peak power density and long‐term stability were 259.1 mW mg^−1^ and 80 h, respectively, which is better than those of commercial Pt/C (177.5 mW mg^−1^, 30 h). The in‐depth insights into the strain‐catalytic performance relationship and monomicelle‐directed synthesis provide a novel strategy for designing and fabricating electrocatalytic materials with optimized spatial organization and targeted functionalities.

## Conflict of Interest

The authors declare no conflict of interest.

## Supporting information

Supporting Information

## Data Availability

The data that support the findings of this study are available in the supplementary material of this article.
