# Supplementary material for: Monomicelle‐Directed Engineering of Strained Carbon Nanoribbons as Oxygen Reduction Catalyst
Source: Adv Sci (Weinh). 2023 Jun 29;10(25):2302930. doi: 10.1002/advs.202302930 (PMC10477895; doi:10.1002/advs.202302930)
Supplement: Supplementary file 1 — Supporting Information [file ADVS-10-2302930-s001.pdf]

ADVANCED SCIENCE
Open Access

Supporting Information

for *Adv. Sci.*, DOI 10.1002/advs.202302930

Monomicelle-Directed Engineering of Strained Carbon Nanoribbons as Oxygen Reduction Catalyst

*Dongping Xue, Yingying Guo, Bang-An Lu, Huicong Xia, Wenfu Yan, Dongfeng Xue, Shichun Mu and Jia-Nan Zhang**

Supporting Information

Monomicelle-directed Engineering of Strained Carbon Nanoribbons as an Oxygen Reduction Catalyst

Dongping Xue,[a] Yingying Guo,[a] Bang-An Lu,[a] Huicong Xia,[a] Wenfu Yan,[d] Dongfeng Xue,[b] Shichun Mu,[c] Jia-Nan Zhang[*,a]

[a] School of Materials Science and Engineering, Zhengzhou University, Zhengzhou 450001, P. R. China

[b] Multiscale Crystal Materials Research Center, Institute of Advanced Materials Science and Engineering, Shenzhen Institute of Advanced Technology, Chinese Academy of Science, Shenzhen, 518055, P. R. China

[c] State Key Laboratory of Advanced Technology for Materials Synthesis and Processing, Wuhan University of Technology, Wuhan 430070, PR China

[d] State Key Laboratory of Inorganic Synthesis and Preparative Chemistry, Jilin University, Changchun, Jilin, 130012 China

*Corresponding authors

E-mails: zjn@zzu.edu.cn (J. N. Zhang)

1. Materials and Methods

Materials

All chemicals were purchased and used without further purification. Triphenylphosphine (TPP), melamine (M) and ethanol were obtained from Sinopharm Chemical Reagent Co., Ltd. Pluronic P123 (P123, PEO-PPO-PEO, Mw = 5800), Nafion (5.0 wt%) and RuO_2 were purchased from Sigma-Aldrich. All chemicals were used as received without any further purification. Pt catalyst (20% Pt supported on Vulcan XC-72 carbon) was obtained from Johnson Matthey. Deionized water was used in all experiments.

Methods

Materials synthesis

Synthesis edge defect-rich curved nitrogen (N)-doped carbon nanoribbons (CNRs-C)

To prepare the precursor slurry, TPP, M (melamine) and P123 (PEO-PPO-PEO, Mw = 5800 g mol^{-1}) were dissolved in 30 ml cohol/H_2O under stirring with the mole ratio of 25 M: 1 TPP: 1 P123 at 60 ^oC for 30 min and dried in an oven at 80 ^oC, and then pyrolyzed at 550 ^oC for 2 h under an Ar atmosphere, g-C_3N_4 samples were obtained. Afterwards, the sample by carbonized at 1000 ^oC for 6 h under an Ar atmosphere and then was followed to cool naturally to room temperature, named CNRs-C.

Synthesis edge defect-rich smooth nitrogen (N)-doped carbon nanoribbons (CNRs-S)

The preparation method of CNRs-S was almost consistent with that of N-GNRs, except that reduceing the amount of P123 (0.5 mmol) added in the precursor slurry.

Synthesis of carbon nanosheets rich in edge defects (CNRs)

The preparation method of CNRs was almost consistent with that of CNRs-C, except that removing P123 in the precursor slurry.

Materials characterizations

The morphology of the samples was studied by field-emission scanning electron microscope (FE-SEM, JEORJSM-6700F) and transmission electron microscope (TEM, FEI Tecnai G2 20) with an accelerating voltage of 200 kV. Powder X-ray diffraction (XRD) patterns were collected using a Y-2000X-ray Diffractometer using copper Kα radiation (λ=1.5406 Å) at 40 kV, 40 mA. The N_2 adsorption/desorption curve was determined by BET measurements using a Micromeritics ASAP 2020 surface area analyzer. The X-ray photoelectron spectroscopy (XPS) measurements were performed with an ESCA LAB 250 spectrometer using a focused monochromatic Al Kα line (1486.6 eV) X-ray beam with a diameter of 200 μm.

Pair Distribution Function (PDF) measurements and Analysis

X-ray absorption spectroscopy (XAS) experiments were performed at Taiwan Photon Source 20A beamline. X-ray total scattering data were measured at BL17b beamline in energy state of 20keV (0.6199 Å) of National Facility for Protein Science (NFPS), Shanghai Synchrotron Radiation Facility (SSRF). The 2D XRD image was first integrated to obtain 1D total scattering intensity I(Q) calibration by LaB6 celebrant by Dioptas 0.5.2 package.[1]Additional scattering measurements from kapton capillary were performed in the same conditions for background subtraction. Then the reduced pair distribution function G(r) was obtained through Fourier transform total scattering structure function S(Q) derivate from I(Q) by PDFgetX3[2, 3].

The following G(r) equation was used:

$$\mathrm{G}(r) = \frac{2}{\pi}\int_0^\infty Q(S(Q)-1)\sin(Qr)dQ$$

The PDF Rietveld refinement was conducted by PDFgui.[3] The initial structural model were built based on soft X-ray XANES spectra and DFT calculation structural model. The following parameters including the scale factor, the cell parameters, the atomic positions except particular positions, the isotropic atomic displacement parameters(Biso), and the dynamic correlation factor (delta2) were refined during the PDF Rietveld refinement.

PDF Rietveld refinement Crystal structure data:

Crystal structure data from PDF Rietveld refinement for N-GNRs, space group *P*1 (no. 1), a = 14.6075 Å, b = 15.00 Å, c = 24.8058 Å, α = 91.28°, β = 89.85°, γ = 91.11°, V = 5432.8719 Å^3, χ_{sq} =185.778, Reduced χ_{sq} = 0.0531;Rw= 0.0512.

Electrocatalytic measurement

A CHI 760E electrochemical workstation (CH Instruments) was used to measure the electrocatalytic activities towards ORR. All the electrochemical measurements were conducted in a three-electrode configuration. The potential, measured against an Ag/AgCl electrode, was converted to the potential versus the reversible hydrogen electrode (RHE). $E_{RHE}=E_{Ag/AgCl}$ + 0.059 pH + 0.197. To prepare the working electrode, 2 mg each of the samples was dispersed in 336μL Isopropanol, 144 μL deionized water and 20 μL of 5 wt% Nafion aqueous solution under sonication. Thereafter, 5 μL of the obtained homogeneous catalyst ink was dropped onto a mirror polished glassy carbon electrode. Pt/C (20 wt%) electrode was prepared by using the same procedure. 0.1 M KOH/0.5 M H_2SO_4 aqueous solutions saturated with oxygen were employed as the electrolyte for ORR. The details for calculation of electron transferred number for ORR is given later.

The calculation of electron transferred number for ORR. On the basis of the RDE data, the electron transfer number per oxygen molecule for oxygen reduction can be determined by Koutechy-Levich equation[4, 5]:

$$\frac{1}{J}=\frac{1}{J_L}+\frac{1}{J_K}=\frac{1}{B\omega^{0.5}}+\frac{1}{J_K} \quad (1)$$

$$B=0.62nFC_0(D_0)^{2/3}\nu^{-1/6} \quad (2)$$

$$J_K=nFkC_0 \quad (3)$$

Where J is the measured current density and is the electrode rotating rate (rad s^{-1}). B is determined from the slope of the Koutechy-Levichi (K-L) plot based on Levich equation (2). J_L and J_K are the diffusion- and kinetic-limiting current densities, n is the transferred electron number, F is the Faraday constant (F = 96485 C mol^{-1}), C_0 is the O_2 concentration in the electrolyte (C_0 = 1.26 × 10^{-6} mol cm^{-3}), D_0 is the diffusion coefficient of O_2 in 0.1 M KOH (D_0 = 1.93 × 10^{-5} cm^2 s^{-1}), and v is the kinetic viscosity (v = 0.01009 cm^2 s^{-1}).

or the RRDE measurements, catalyst inks and electrodes were prepared by the same method as for RDE. The disk electrode was scanned at a rate of 5 mV s^{-1}, and the ring potential was constant at 1.3 V vs RHE. The HO_2^-% and transfer number (n) were determined by the followed equations[6]:

$$HO_2^-\% = 200\times\frac{I_R/N}{I_D+I_R/N} \quad (4)$$

$$n=4\times\frac{I_D}{I_D+I_R/N} \quad (5)$$

where I_D is disk current, I_R is ring current, and N is current collection efficiency of the Pt ring. N was determined to be 0.40.

All electrochemical test voltages were not iR compensated.

MEA Preparation and Fuel Cell Testing

Catalysts were mixed with Nafion, isopropyl alcohol, and water by ultrasonication for 1 hour to form homogeneous ink. The ink has then sprayed the ink onto Nafion 211 membrane at 80°C. Fuel cell testing was performed in a single cell using a commercial fuel cell test system (SMART2). The MEA was sandwiched between two graphite plates with single serpentine flow channels. The cell was operated at 80°C with a back pressure of 200 kPa. Pure hydrogen and air/oxygen, with 100% relative humidity (RH), were supplied to the anode and cathode at a gas flow rate of 200-300 sccm.

All-solid-state Zn–air battery assembly

A clean zinc foil (~99.98 % metal basis) was used as anode and a solid polymer electrolyte as separator. To form the solid polymer electrolyte, polyvinyl alcohol powder (5 g) was dissolved in 50 mL of deionized water at 95 ℃ about 2 h to form a homogeneous viscous solution, and then by dropping join 5 mL 18 M KOH into above-mentioned solution, followed by casting on a culture dish to form a thin polymer film. The film was then frozen for 2 h and thawed for 4 h before used. To package the battery, a thin layer of the catalyst and zinc foil were placed on opposite sides of the polymer electrolyte film followed by placing nickel foam on the ambient dried catalyst layer as current collector. The catalyst loading was ~0.5 mg cm^{-2}. No inert atmosphere or glove-box is required for the packaging.

Computational Section

First principles calculations were carried out within the density functional theory framework.[7] The projector-augmented wave (PAW) method[8, 9] and the generalized gradient approximation (GGA)[10] for the exchange-correlation energy functional, as implemented in the Vienna *ab initio* simulation package (VASP)[11] were used. The GGA calculation was performed with the Perdew-Burke-Ernzerhof (PBE)[12] exchange-correlation potential. A plane-wave cutoff energy of 400 eV was used.

All atoms were fully relaxed with a tolerance in total energy of 0.1 meV, and the forces on each atom were less than 0.02 eV/Å. The van der Walls interactions were included by DFT-D2[13] method. The periodic model and the nanoribbon model were used to simulate the ORR reaction happened on N-doped graphene with a certain strain and rich edge defects. The details of each catalyst can be seen in the Supporting Information.

For ORR, the Gibbs free energy is calculated by the generally reported four electrons process:

(1) $O_2 + * \Rightarrow O_2*$

(2) $O_2* + H_2O\ (l) + e^- \Rightarrow OOH* + OH^-$

(3) $OOH* + e^- \Rightarrow O* + OH^-$

(4) $O* + H_2O\ (l) + e^- \Rightarrow OH^- + OH*$

(5) $OH* + e^- \Rightarrow OH^- + *$

The change in free energy (ΔG) of per reaction step is calculated as:

$$\Delta G = \Delta E + \Delta ZPE - T\cdot\Delta S + \Delta G_U + \Delta G_{pH}$$

Where ΔE is the change of the total reaction energy obtained from DFT calculation, ΔZPE is the change of the zero-point energy, T is the temperature (300K), and ΔS is the change of the entropy. ΔG_U=-eU, here U is the potential at the electrode and e is the transferred charge. $\Delta G_{pH}=k_B\cdot T\times \ln 10\times pH$ where k_B is the Boltzmann constant and T =300 K. In this work, the influence of PH was neglected. The free energy of O_2 is obtained from the reaction $O_2+2H_2 \rightarrow 2H_2O$, which is 4.92 eV at 300K and pressure of 0.035 bar. The free energy of OH^- is defined as $G(H_2O) - G(H^+)$, and the free energy of H^+ is equal to $1/2H_2$. The entropies of molecules (including O_2, H_2 and H_2O, etc.) in the gas (or liquid) phase are taken from the "CRC Handbook of Chemistry and Physics". The free energy of H+ ions has been corrected by the concentration dependence of the entropy[14]:

$G(\mathrm{pH}) = -kT\ln H^{+} = kT\ln 10 * \mathrm{pH}$

(0.059526 for 0.1M $HClO_4$; 0.773844 for 0.1M KOH).

For nanoribbon model, the armchair and zigzag graphene nanoribbon were used in the calculation.[15]

For periodic model, a 8×8 graphene supercell (19.754×19.754) was first constructed. This model was used to simulate the ORR reaction happened on N-doped graphene with strain and rich edge defects. N doped graphene with different strain degree was used as catalyst for ORR reaction was also calculated for comparation. In the calculation, the vacuum layer is 12 Å and a 1x1x1 K-pointes was used.

2. Supplementary Figures and Tables

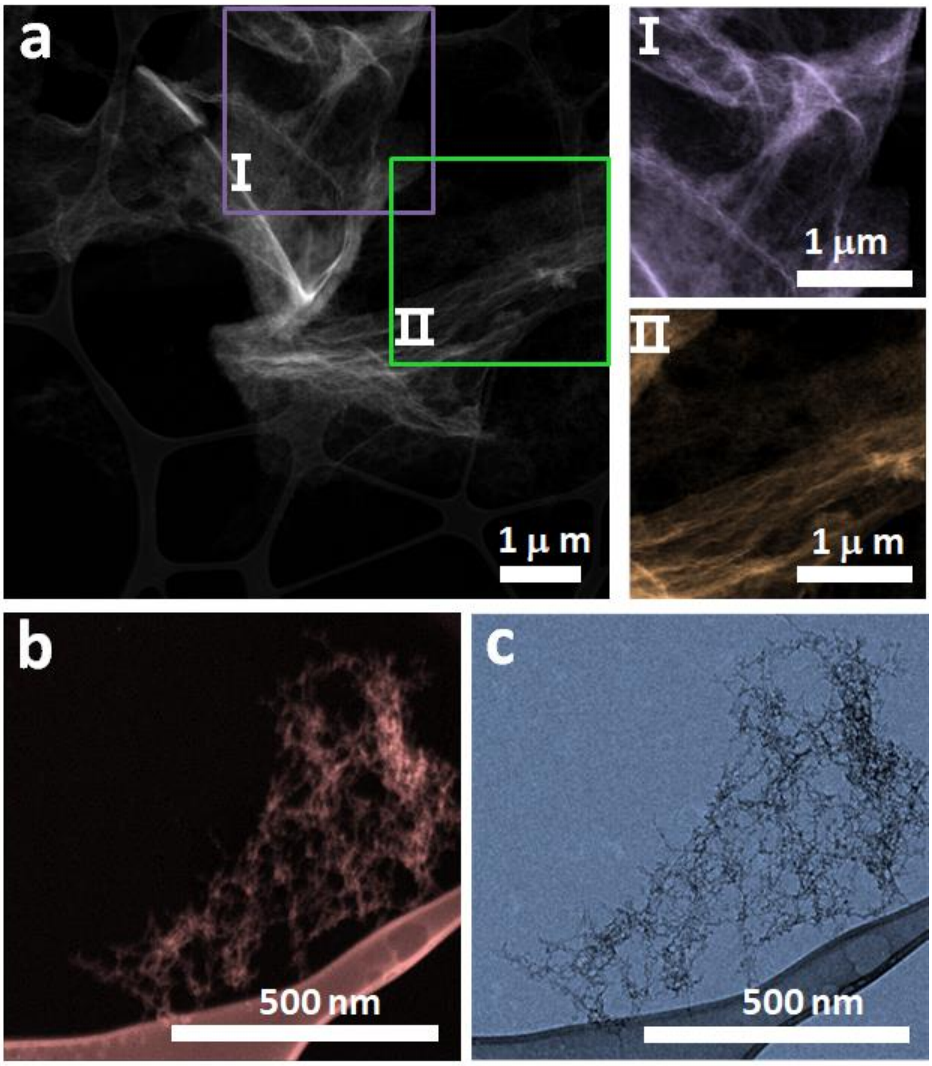

Figure S1. (a,b) Different magnification HADDF-STEM images, and (c) TEM image corresponding to b of the defective carbon nanoribbons (CNRs-C).

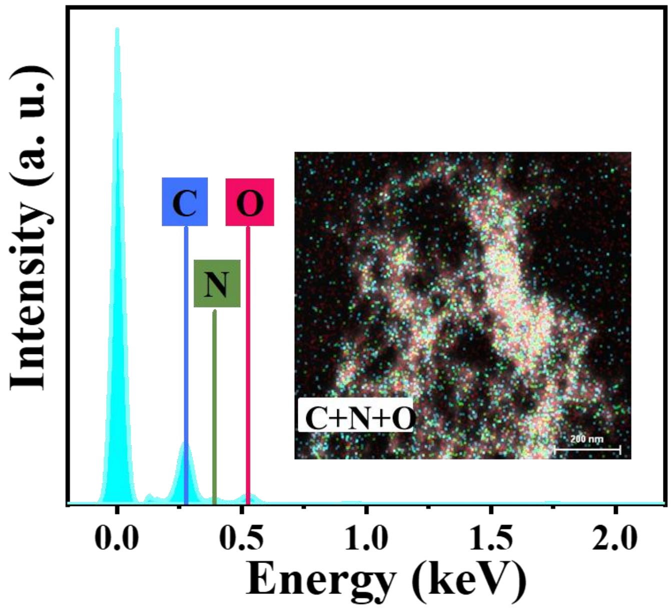

Figure S2. HAADF-STEM image of the CNRs-C and corresponding element maps showing the distribution of the C, N and O.

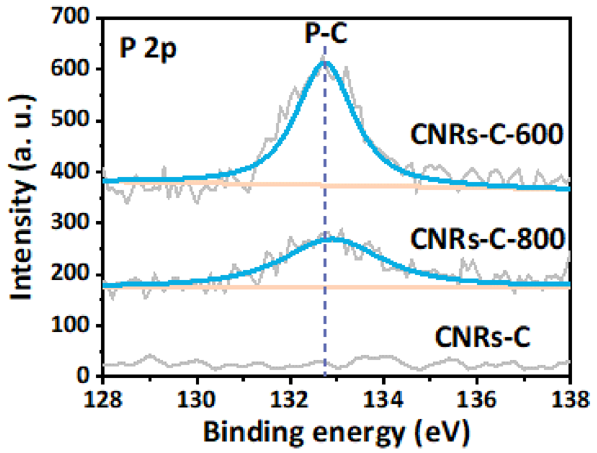

Figure S3. High-resolution spectra of P2p.

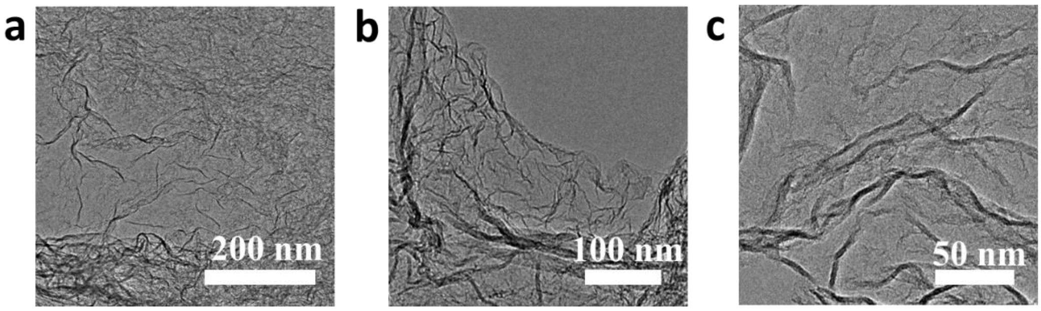

Figure S4. Different magnification (a,b,c) TEM images of CNSs.

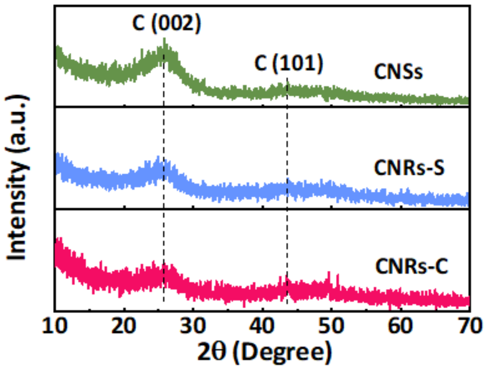

Figure S5. XRD patterns of CNSs,CNRs-S and CNRs-C.

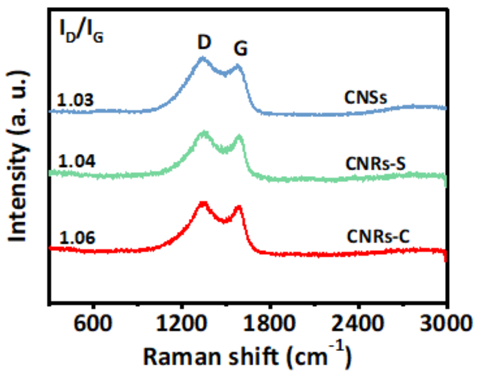

Figure S6. Raman spectra of CNSs,CNRs-S and CNRs-C.

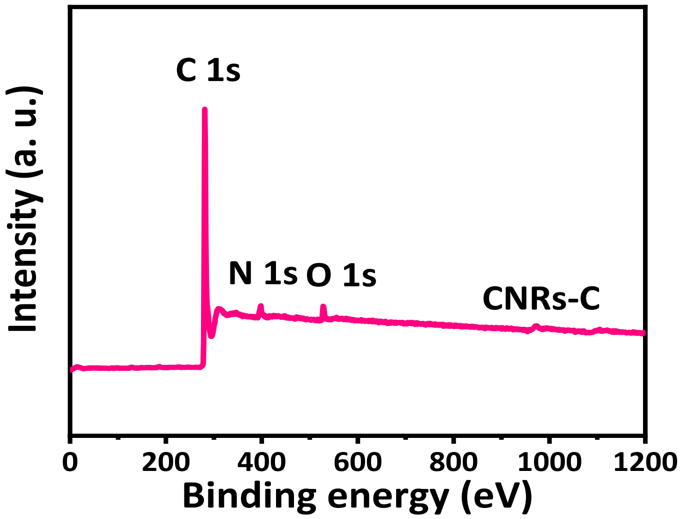

Figure S7. Survey XPS spectrum of CNRs-C.

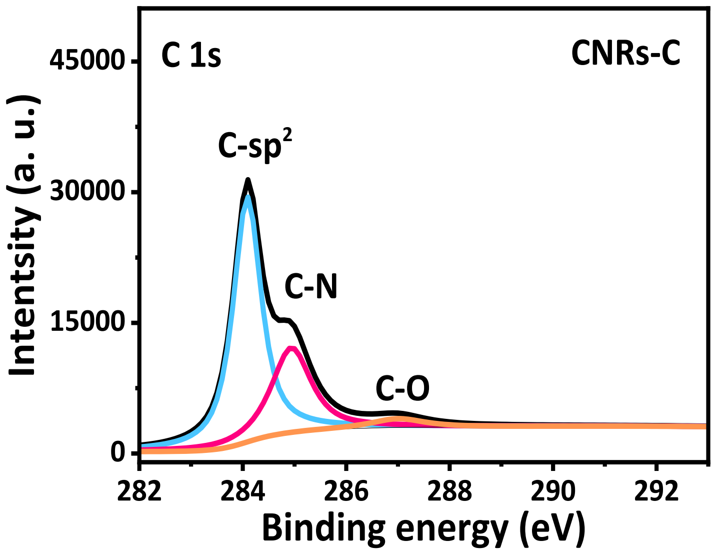

Figure S8. High-resolution spectra of XPS C1s of CNRs-C.

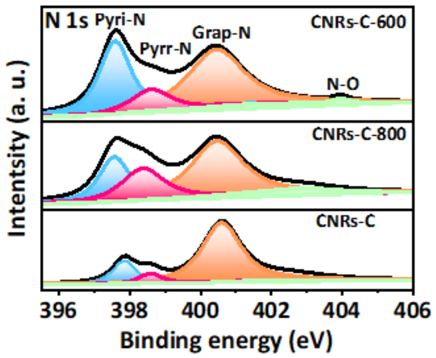

Figure S9. High-resolution spectra of N1s

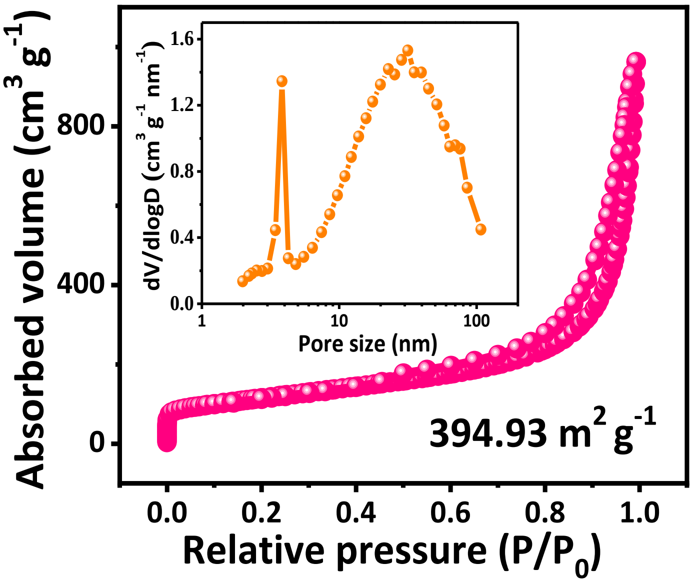

Figure S10. N_2 adsorption-desorption isotherm and the pore size distribution of CNRs-C.

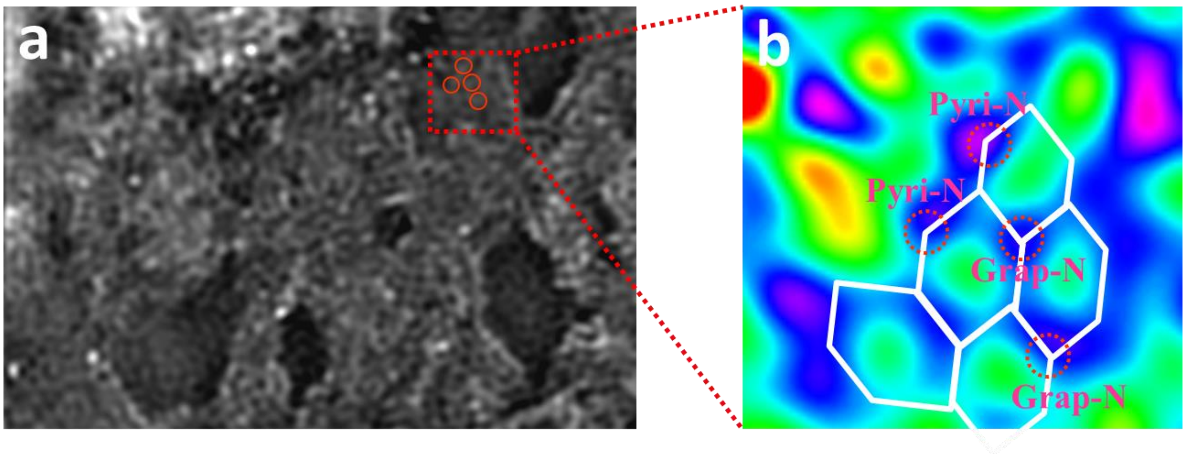

Figure S11. (a) The HAADF-STEM image of CNRs-S. (b) Expanded image of the dotted box in h, presenting the Pyri-N and Grap-N sites at the edge, respectively.

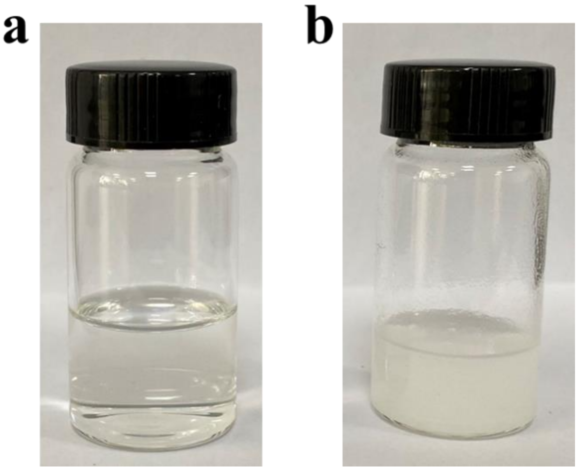

Figure S12. (a) The ethanol/H_2O solution including Melamine (M), Pluronic P123, Triphenyl phosphorus (TPP), before the evaporation at 80 ºC. (b) The composite micelles obtained by the evaporation of the mixed solution shown in (a).

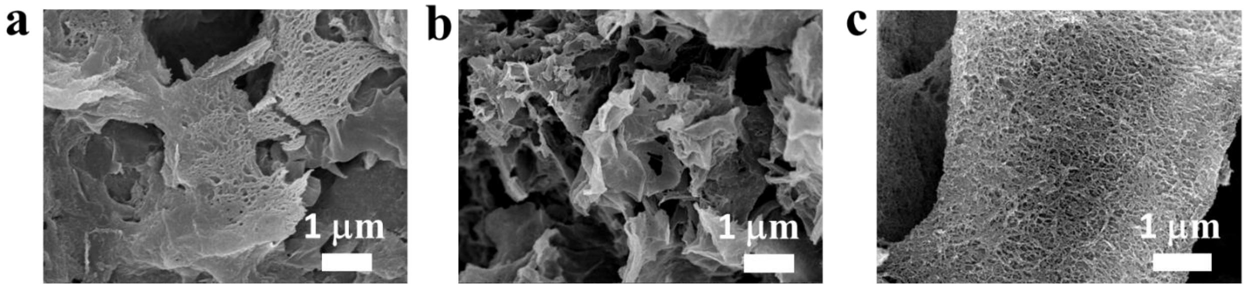

Figure S13. Different magnification SEM images of (a) CNRs-C-600, (b) CNRs-C-800, and CNRs-C(c).

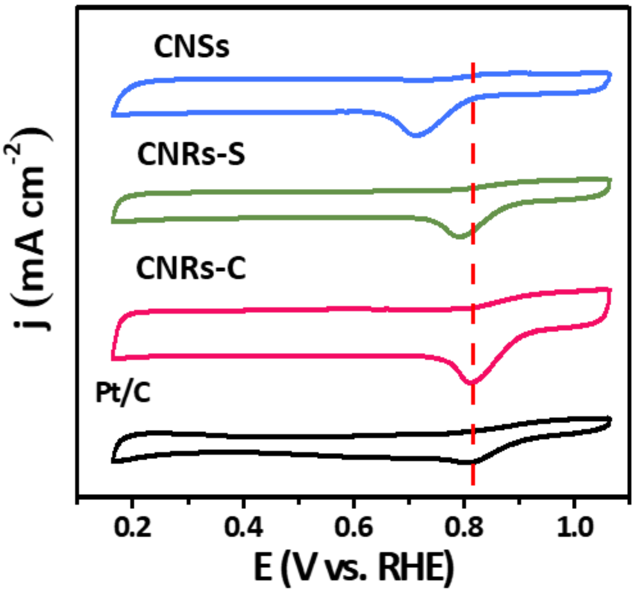

Figure S14. CVs of GNSs, CNRs-S, CNRs-C catalysts and Pt/C catalysts in O_2-saturated solution at a scanning rate of 50 mV s^{-1} in 0.1 M KOH.

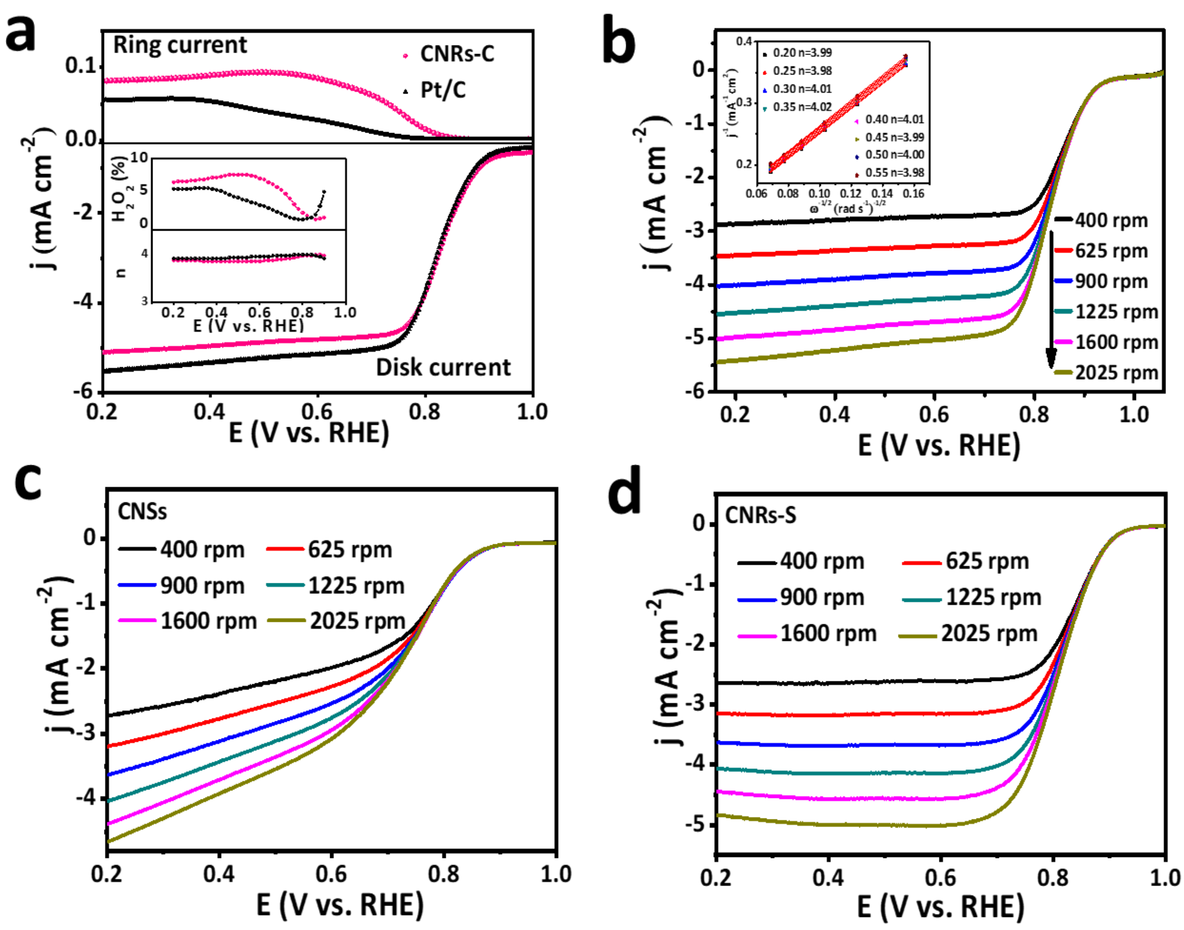

Figure S15. (b-d) LSV curves of CNRs-C (inset is the K-L plots and electron transfer number (n)), CNSs and CNRs-S catalysts in O_2-saturated 0.1 M KOH solution. Scan rate is 5 mV s^{-1}.

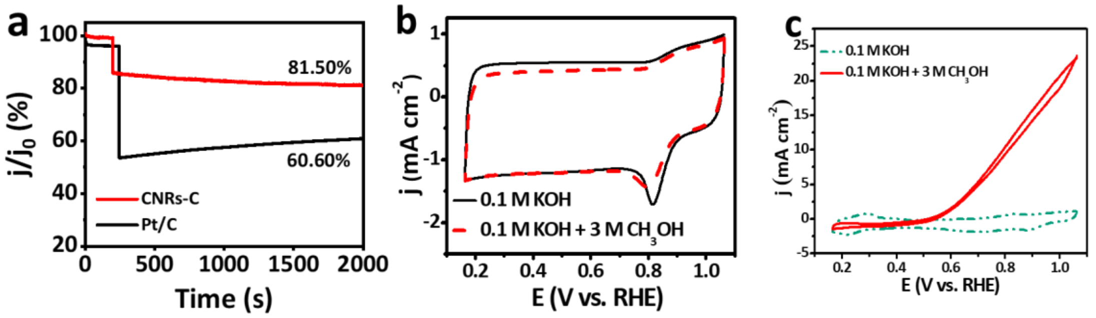

Figure S16. (a) Chronoamperometric response of CNRs-C and Pt/C catalyst at 0.75 V (*vs*. RHE) after the injection of 30.3 mL of CH_3OH into 219.7 mL of 0.1 M KOH. CV profiles of (b) CNRs-C and (c) Pt/C (black and red curves indicate CV curves recorded in O_2-saturated 0.1 M KOH and O_2-saturated 0.1 M KOH + 3 M CH_3OH solution, respectively).

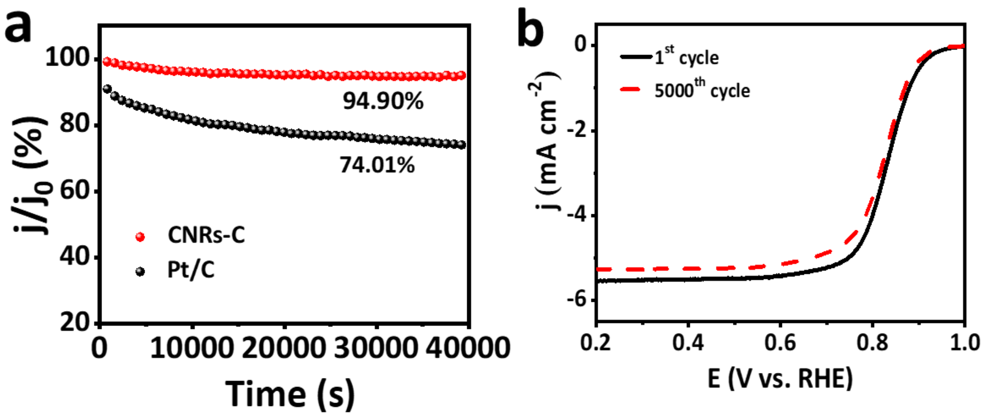

Figure S17. (a) Durability tests: chronoamperometric response of CNRs-C and Pt/C catalyst at 0.75 V (*vs.* RHE). (b) ORR polarization curves (1600 rpm) of Pt/C before and after 5,000 cycles.

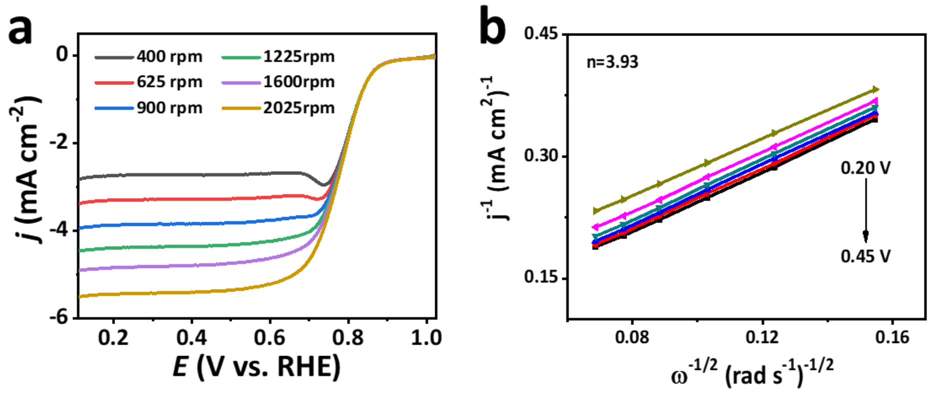

Figure S18. (a) LSV curves of CNRs-C and (b) electron transfer number (n) in O_2-saturated 0.5 M H_2SO_4 solution.

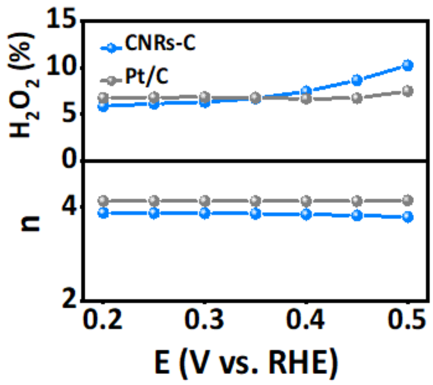

Figure S19. H_2O_2 yield and electron transfer number (n) for CNRs-C and Pt/C in O_2-saturated 0.5 M H_2SO_4.

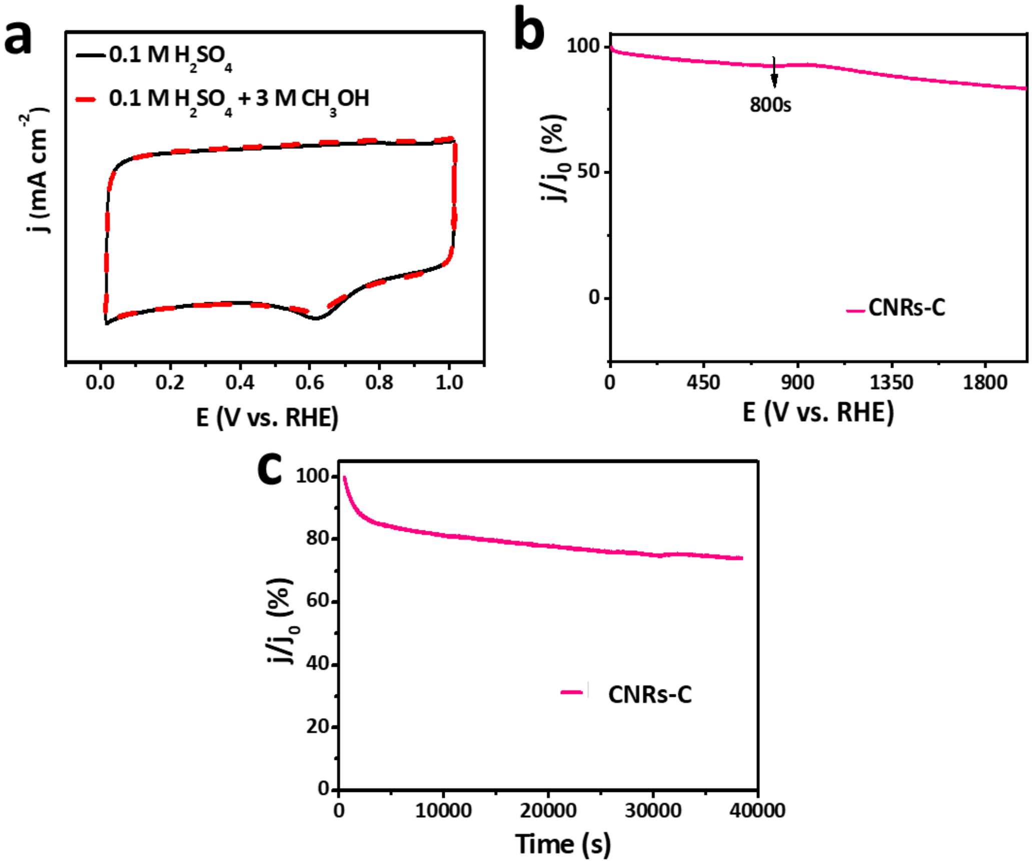

Figure S20. Electrochemical methanol tolerance tests: (a) CV profiles of CNRs-C with and without CH_3OH (b) Chronoamperometric response of CNRs-C at 0.75 V (*vs*. RHE) after the introduction of 30.3 mL of CH_3OH into 219.7 mL of 0.5 M H_2SO_4 solution. (Durability tests: (c) Chronoamperometric response of CNRs-C at 0.75 V (*vs*. RHE).

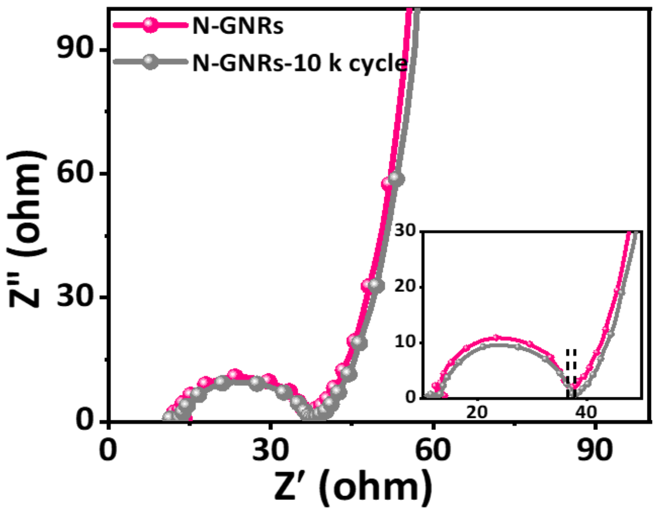

Figure S21. The electrochemical impedance spectroscopy (EIS) of N-GNRs before and after cycling at the potential of 1.0 V vs. RHE.

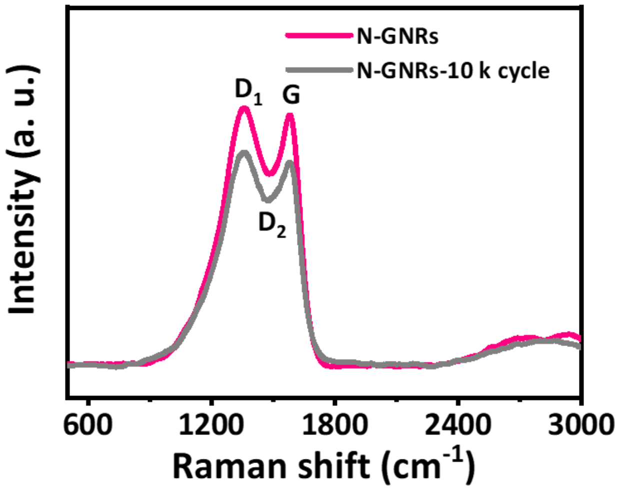

Figure S22. Raman spectra of N-GNR before and after cycling.

We further investigated the reasons for the slight decrease in the stability of N-GNRs. According to previous reports,[16-18] for carbon-based materials used in ORR, the oxidation corrosion of carbon is the origin for the decrease in stability, and the main reasons for causing or aggravating carbon oxidation corrosion are as follows: 1. Reaction environment containing O_2; 2. Oxygen-containing intermediate and by-product H_2O_2 formed during ORR; 3. High temperature or high pressure conditions (mainly in the start-stop phase of fuel cell test). The first two reasons are unavoidable in ORR half-cell test, therefore, we verify whether the conductivity of N-GNRs catalyst decreases before

and after the stability test (10,000 cycles) by impedance test. As shown in **Figure S21**, the oxidative corrosion of carbon resulted in a slight decrease in the electronic conductivity of the catalyst as expected. Next, we investigated the carbon structures of the N-GNRs catalysts before and after the stability test by Raman spectroscopy. As shown by **Figure S22**, the D_1 (1353 cm^{-1}) and G (1577 cm^{-1}) bands of the samples after cycling were narrowed, as well as a significant decrease of the intensity of the D_2 (1476 cm^{-1}) band, demonstrating that carbon corrosion occurred during the ORR cycle, leading to a slight decrease in activity.

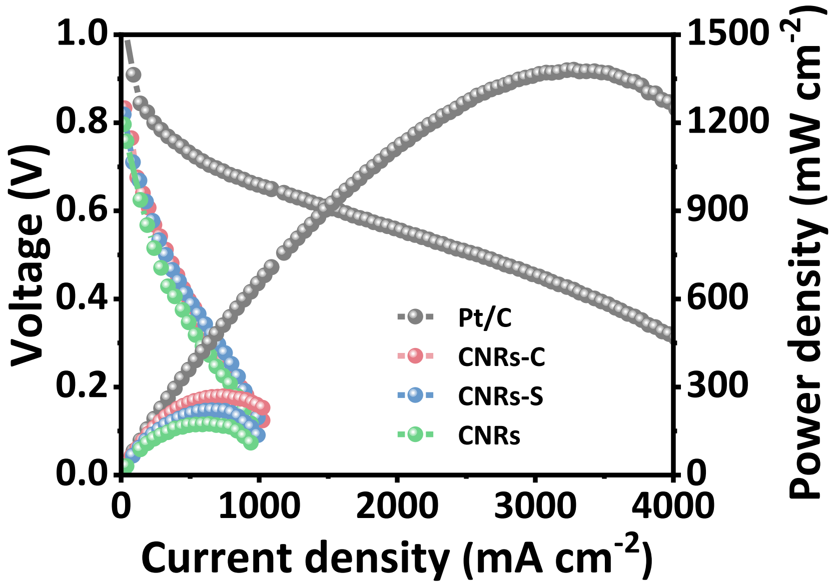

Figure S23. Polarization and power density curves of Pt/C, CNRs-C, CNRs-S and CNRs under 2 bar H_2-O_2.

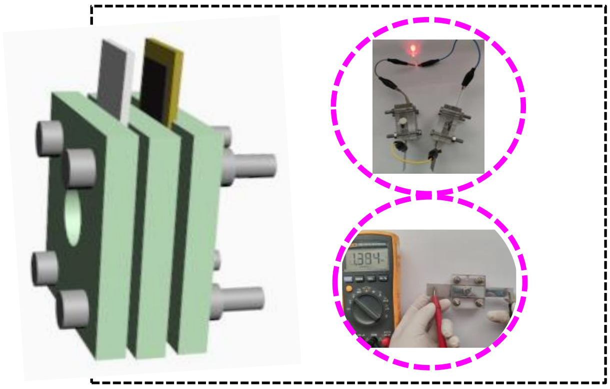

Figure S24. Photograph of liquid batteries with an open circuit voltage of 1.394V and LED powered by two liquid Zn-air batteries with the CNRs-C as air-cathode connected in series.

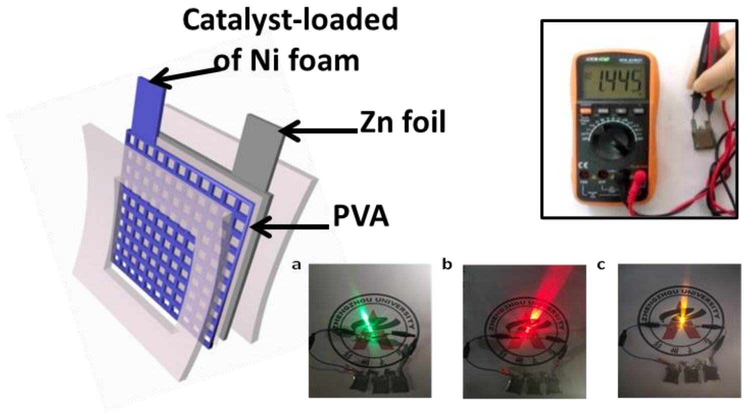

Figure S25. Schematic for fabrication of all-solid-state zinc-air battery. Photograph of all-solid-state zinc-air battery displaying a measured open circuit voltage of ~ 1.445 V. Photograph of a lighted LED powered (Left o right be blue, red, and yellow) by three batteries.

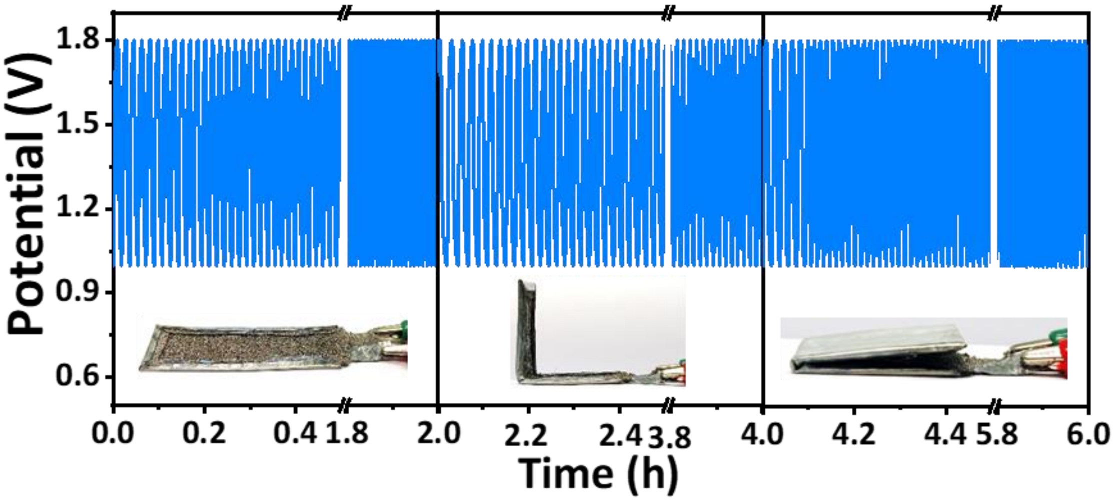

Figure S26. Galvanostatic discharge-charge cycling curve at 2 mA cm^{-2} for the all-solid-state rechargeable zinc-air battery, applying bending strain (as depicted by the inset images) every 2 h.

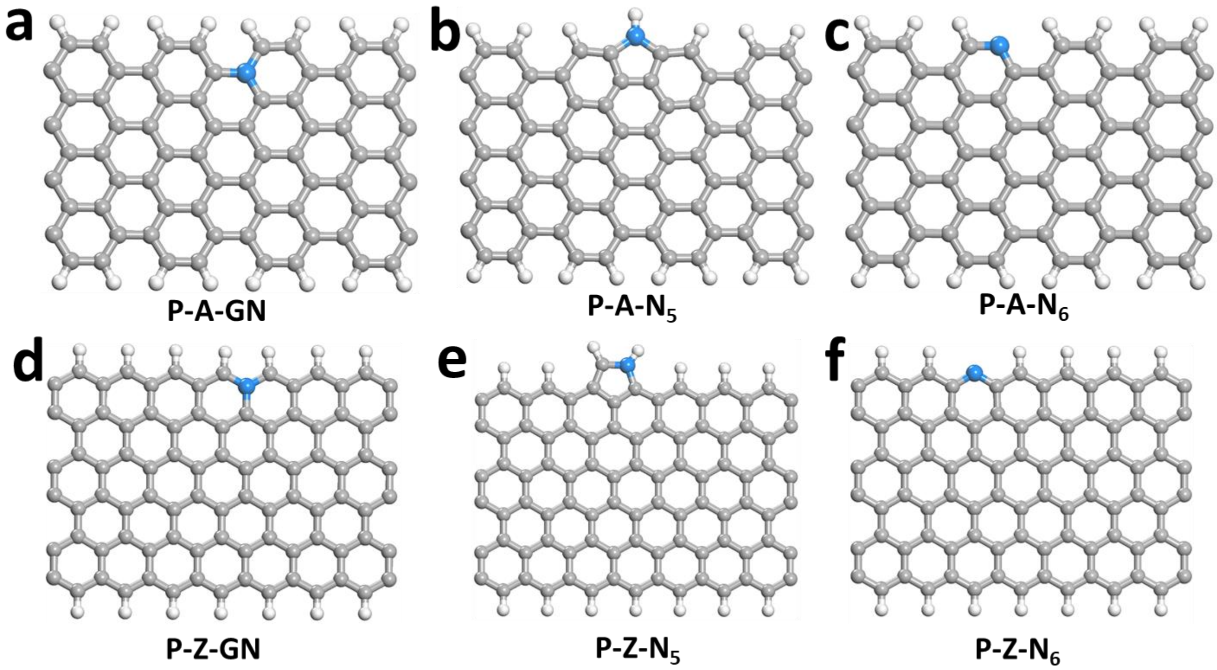

Figure S27. Atomic structures of different planar N-doped C (percentage represents the degree of curve). The gray, blue, and white balls represent C, N, and H atoms, respectively.

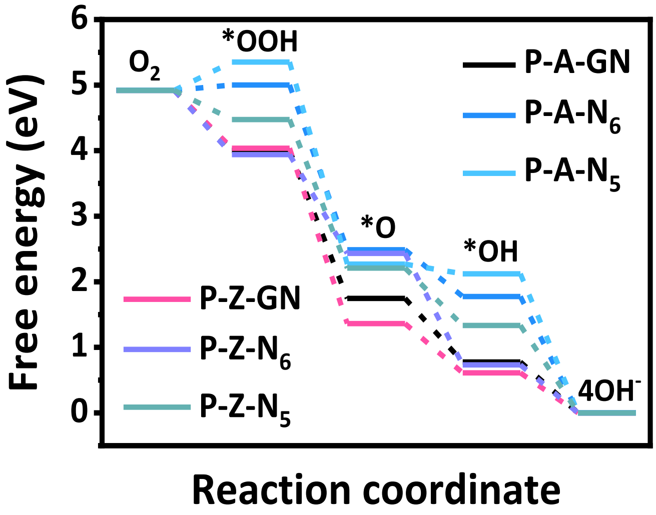

Figure S28. Free energy diagrams of planar N-doped C structures.

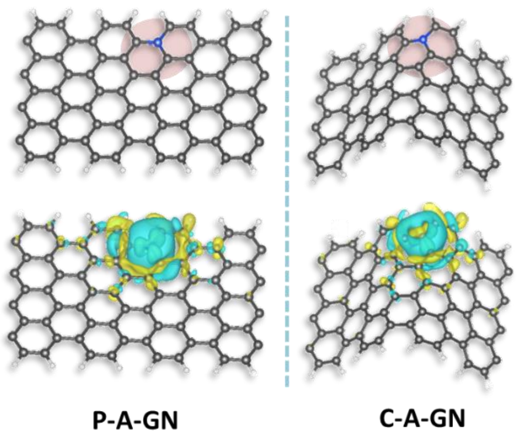

Figure S29. Geometric structure and charge distribution of the fully relaxed and CNRs-C. Here, the gray, blue, and white balls represent C, N, and H atoms, respectively. The charge distribution is shown on the isosurface of 0.005 eÅ^{-3} with yellow representing charge accumulation and gree representing charge depletion.

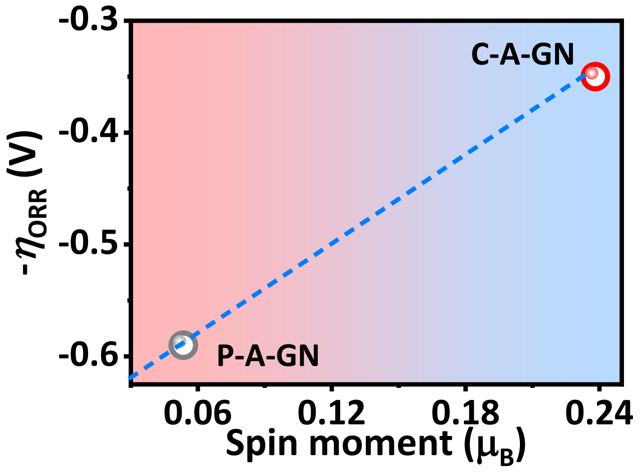

Figure S30. Plot of ORR overpotential *vs.* spin moment on the active carbon atom.

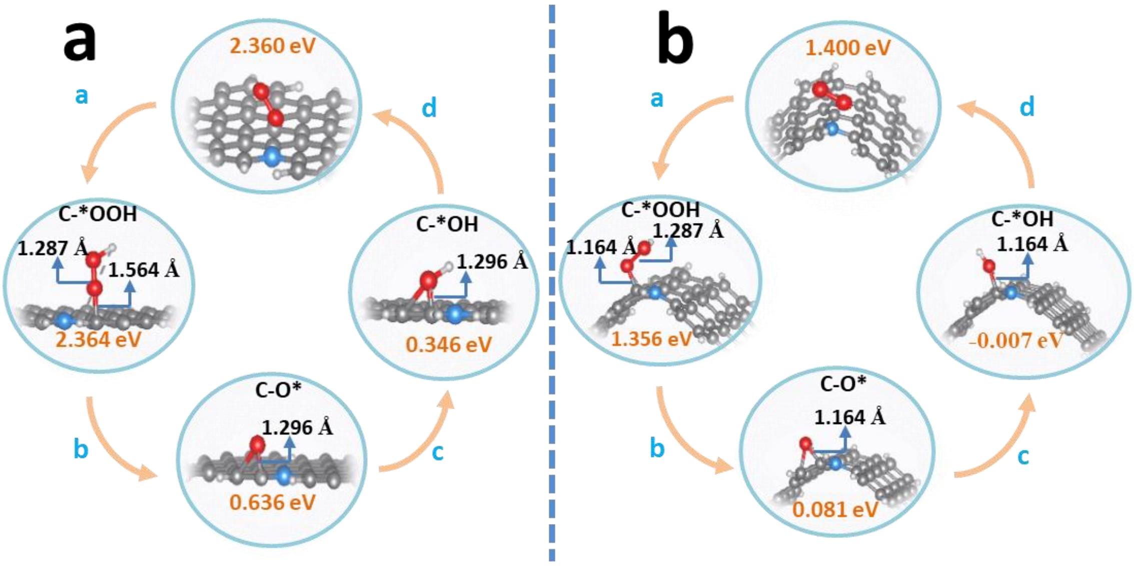

Figure S31. Optimized atomic structures for the main process of an ORR: (a) P-A-GN and (b) C-A-GN.

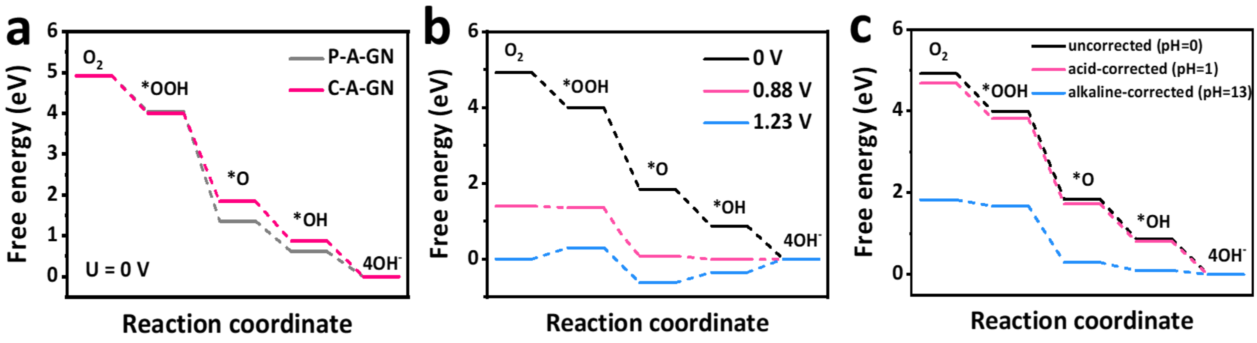

Figure S32. (a) Free energy diagrams of P-A-GN and C-A-GN (b) The pathways for C-A-GN are summarized at U =0 V, 0.88 V, and 1.23 V, respectively. (c) pH-corrected free energy diagram of the C-A-GN. The acid-corrected one does not significantly change the free energy diagram, while the alkaline-corrected one brings a noticeable change. However, the pH correction has no impact on the ORR trend under the same calculation model. Therefore, the change of electrolyte does not change the conclusion of this research work: E C-A-GN model possess the highest theoretical ORR activity.

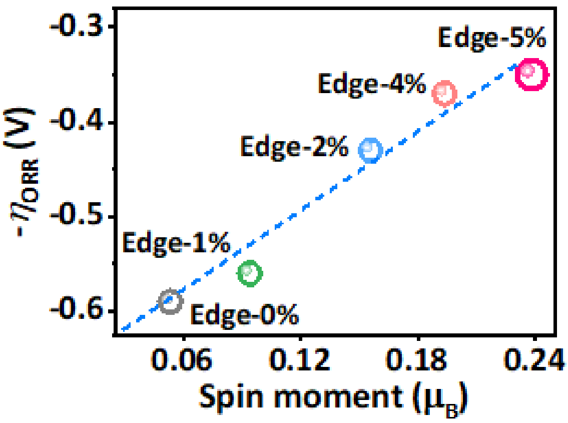

Figure S33. Plot of ORR overpotential *vs.* spin moment on the active carbon atom with different curvature structures.

To further reveal the relationship between curvature and catalytic activity, based on DFT, we calculated the ORR limit potentials (U_L), charge accumulation number, spin moment (μ_B) and oxygen reduction reaction overpotentials (η_{ORR}) on the carbon active site in different curvature structures of 0%, 1%, 2%, 4% and 5% respectively, as shown in **Figure S33**, **Table S9**, and **Table S10**.

- As shown in Table S9, U_L on these catalysts follows the order: Edge-0% (0.64 V) < Edge-1% strain (0.67 V) < Edge-2% strain (0.80 V) < Edge-4% strain (0.86 V) < Edge-5% strain (0.88 V), suggesting that Edge-5% strain serves the most thermodynamically favourable site for ORR among all the catalysts.
- As shown in Table S10, the charge distribution analysis suggests that Edge-5% strain can favour the electrons transferred from the carbon atom site to the adjacent N atom, resulting in a significant charge accumulation on carbon, which is beneficial to improve the adsorption and desorption of reaction intermediates under the strain effect.
- As shown in Figure S33, the carbon atoms in Edge-0% are not magnetic with negligible spin moment (μ_B) (0.053 μ_B), however, this value dramatically increases to 0.094, 0.156, 0.194, and 0.238 μ_B for 1%, 2%, 4%, and 5% curved structures, respectively. It is found that there is a linear correlation between the spin moment and the curvature, which is responsible for the accelerated ORR.

Undoubtedly, the significantly increased limit potential and reduced energy barrier in the reduction reaction ensure that the high strain carbon nanoribbons have significantly enhanced ORR activity.

Tables

Table S1. XPS results analysis for the prepared samples (at %).

Sample Name	C (Atom %)	O (Atom %)	N (Atom %)	P (Atom %)
CNRs-C-600	74.17	7.8	17.61	0.41
CNRs-C-800	81.19	10.16	8.58	0.07
CNRs-C	87.72	8.24	3.56	0.00

Table S2. The XPS content of N species (%).

**Sample Name**	**pyridinic N**	**pyrrolic N**	**graphitic N**	**nitrogen molecules and/or oxides**
**CNRs-C**	16.02	6.8	66.68	10.50

Table S3. Surface and pore related parameters from N_2 adsorption isotherms of samples.

Sample Name	BET surface area (m^2 g^{-1})	Pore size (nm)
CNSs	370.29	3.82/32.2
CNRs-S	414.57	3.8/32.1
CNRs-C	394.93	3.84/31.62

Table S4. Comparison of the oxygen electrode activities of the recently reported highly active ORR catalysts. $E_{1/2}$ is the ORR half-wave potential; E_{onset} is the ORR onset potential; All data are observed in 0.10 M KOH electrolyte.

**Catalyst**	***E*$_{1/2}$ (V vs. RHE)**	***E*$_{onset}$ (V vs. RHE)**	**Catalyst loading (mg cm^{-2})**	**Ref.**
**CNRs-C**	**0.84**	**0.95**	**0.10**	**This work**
D-CM	0.81	-	0.30	Chem 2020, 6, 2009-2023
PD/N-C	0.833	0.911	0.40	Angew. Chem. Int. Ed. 2019, 58, 3859-3894
GNR@CNT	0.819	0.96	0.398	Nat. Commun. 2018, 9, 3819.
NGM	0.77	0.89	0.25	Adv. Mater. 2016, 28, 6845-6851
NPMC-1000	0.85	0.94	0.15	Nat. Nanotechnol. 2015, 10, 444.

Table S5. Gravimetric activities of various metal-free electrocatalysts compared with the CNRs-C in PEM fuel cells.

Materials	Peak power Density (mW cm^{-2})	Catalyst loading (mg cm^{-2})	O_2-H_2 absolute pressure (bars)	Cell temperature (°C)	Ref.
GO/Nafion	64.38	2	0	60	J. Membr. Sci. 2015, 492, 58
NG	397	1.5	N.A.	50	Int. J. Hydrogen Energy 2013, 38, 9362
N-G-CNT+KB	150	0.5	2.5	80	Sci. Adv. 1, e1400129 (2015)
NG@MMT	320	2	N.A.	N.A.	Angew. Chem. Int. Ed. 52, 11755-11759 (2013)
bNGr	208	4	2.5	80	J. Am. Chem. Soc. 136, 9070-9077 (2014)
GNR@CNT	161	0.25	2	80	Nat. Commun. 2018; 9: 3819
N-GNR@CNT+XC-72	245	3.1	2	80	Nat. Commun. 2018; 9: 3819
DMWNT-H_2SO_4-Ar900	111	1.85	3.5	90	Energy Environ. Sci. 7, 1950-1958 (2014)
**CNRs-C**	**272.7**	**4**	**1**	**80**	**This work**

Table S6. Limiting potentials of ORR reaction of carbon-based structures with planar zigzag (P-Z) and armchair (P-A) edge defects doped with different types of N (pyridine N (N_6), pyrrole N (N_5), and graphite N (G))

	P-A-G	P-Z-G	P-A-N_6	P-Z-N_6	P-A-N_5	P-Z-N_5
limiting potentials (V)	0.64	0.61	-	0.63	-	0.44

Table S7. Average number of electrons gained and lost in the edge graphite N and surrounding C in the charge density difference map of the P-A-GN and C-A-GN.

Element	P-A-GN	C-A-GN
C	-0.3670	-0.4514
N	1.2028	1.2248

Table S8. The reaction position is on C next to N of reaction energetics for the 4-elctron transfer processes during ORR in P-A-GN and C-A-GN, respectively. ΔG, free energy change at T=298 K, pH=13 and U=0 V.

Elementary Reactions	**P-A-GN**	**C-A-GN**
O_2 (g)+*	4.92	4.92
O_2+e^- >OOH*	4.28441	3.9967
OOH*+e^- >O*	1.91667	1.84134
O*+e^- >OH*	0.98661	0.87244
Limit potential (V)	0.64	0.88
Overpotential (V)	0.59	0.35

Table S9. The reaction position is on C next to N of reaction energetics for the 4-elctron transfer processes during ORR in zigzag and armchair nanoribbons, respectively. ΔG, free energy change at T=298 K, pH=13 and U=0 V.

Elementary Reactions	0%	1%	2%	4%	5%	6%	8%
O_2 (g)+*	4.92	4.92	4.92	4.92	4.92	4.92	4.92
O_2+e^- >OOH*	4.28441	4.25036	4.12142	4.0636	3.9967	3.92959	3.87127
OOH*+e^- >O*	1.91667	2.04952	1.99595	1.92586	1.84134	1.74298	1.68393
O*+e^- >OH*	0.98661	1.05877	1.00939	0.94646	0.87244	0.79199	0.72665
Limit potential	0.64	0.67	0.80	0.86	0.88	0.80	0.73
Overpotential (V)	0.59	0.56	0.43	0.37	0.35	0.43	0.5

Table S10. Average number of electrons gained and lost in the edge graphite N and surrounding C in the charge density difference map of the N-GNRs model with different strain degrees.

**Element**	**0%**	**1%**	**2%**	**4%**	**5%**	**6%**	**8%**
C	-0.3670	-0.4518	-0.489	-0.4085	-0.4514	-0.4669	-0.3757
N	1.2028	1.2276	1.2427	1.2144	1.2148	1.2091	1.1687

REFERENCES

[1] C. Prescher, V. B. Prakapenka, *High Pressure Res.* **2015**, 35, 223.

[2] C. L. Farrow, P. Juhas, J. W. Liu, D. Bryndin, E. S. Bozin, J. Bloch, T. Proffen, S. J. Billinge, *J Phys Condens Matter* **2007**, 19, 335219.

[3] P. Juhás, T. Davis, C. L. Farrow, S. J. L. Billinge, *J. Appl. Crystallogr.* **2013**, 46, 560.

[4] Y. Tong, P. Chen, T. Zhou, K. Xu, W. Chu, C. Wu, Y. Xie, *Angew. Chem. Int. Ed.* **2017**, 56, 7121.

[5] Y. Guo, P. Yuan, J. Zhang, Y. Hu, I. S. Amiinu, X. Wang, J. Zhou, H. Xia, Z. Song, Q. Xu, S. Mu, *ACS Nano* **2018**, 12, 1894.

[6] S. Guo, P. Yuan, J. Zhang, P. Jin, H. Sun, K. Lei, X. Pang, Q. Xu, F. Cheng, *Chem. Commun. (Camb.)* **2017**, 53, 9862.

[7] W. Kohn, L. J. Sham, *Physical Review* **1965**, 140, A1133.

[8] E. Torres, T. P. Kaloni, *Comput. Mater. Sci* **2020**, 171.
[9] J. P. Perdew, Y. Wang, *Phys Rev B Condens Matter* **1992**, 45, 13244.
[10] G. Kresse, J. Hafner, *Phys Rev B Condens Matter* **1993**, 47, 558.
[11] G. Kresse, J. Furthmuller, *Phys Rev B Condens Matter* **1996**, 54, 11169.
[12] R. Sassi, R. R. Bond, A. Cairns, D. D. Finlay, D. Guldenring, G. Libretti, L. Isola, M. Vaglio, R. Poeta, M. Campana, C. Cuccia, F. Badilini, *J. Electrocardiol.* **2017**, 50, 776.
[13] J. P. Perdew, K. Burke, M. Ernzerhof, *Phys. Rev. Lett.* **1996**, 77, 3865.
[14] S. Grimme, *J. Comput. Chem.* **2006**, 27, 1787.
[15] G. Henkelman, A. Arnaldsson, H. Jónsson, *Comput. Mater. Sci* **2006**, 36, 354.
[16] K. Kumar, L. Dubau, M. Mermoux, J. Li, A. Zitolo, J. Nelayah, F. Jaouen, F. Maillard, *Angew. Chem. Int. Ed.* **2020**, 59, 3235.
[17] R. Chenitz, U. I. Kramm, M. Lefèvre, V. Glibin, G. Zhang, S. Sun, J.-P. Dodelet, *Energy Environ. Sci.* **2018**, 11, 365.
[18] C. H. Choi, H.-K. Lim, M. W. Chung, G. Chon, N. Ranjbar Sahraie, A. Altin, M.-T. Sougrati, L. Stievano, H. S. Oh, E. S. Park, F. Luo, P. Strasser, G. Dražić, K. J. J. Mayrhofer, H. Kim, F. Jaouen, *Energy Environ. Sci.* **2018**, 11, 3176.
